# Metal-organic framework (MOF) integrated Ti_3_C_2_ MXene composites for CO_2_ reduction and hydrogen production applications: a review on recent advances and future perspectives

**DOI:** 10.3389/fchem.2024.1448700

**Published:** 2024-10-01

**Authors:** Beenish Tahir, Abdulrahman Alraeesi, Muhammad Tahir

**Affiliations:** ^1^ Chemical and Petroleum Engineering Department, UAE University, Al Ain, United Arab Emirates; ^2^ National Water and Energy Research Center, United Arab Emirates University, Al Ain, United Arab Emirates

**Keywords:** titanium carbide (Ti_3_C_2_) MXenes, metal-organic frameworks (MOF), photocatalysis, CO_2_ reduction, hydrogen production, nanosheets

## Abstract

Titanium carbide (Ti_3_C_2_) MXenes due to their structural and optical characteristics rapidly emerged as the preferred material, particularly in catalysis and energy applications. On the other hand, because of its enormous surface/volume ratio and porosity, Metal-organic Frameworks (MOFs) show promise in several areas, including catalysis, delivery, and storage. The potential to increase the applicability of these magic compounds might be achieved by taking advantage of the inherent flexibility in design and synthesis, and optical characteristics of MXenes. Thus, coupling MOF with Ti_3_C_2_ MXenes to construct hybrid composites is considered promising in a variety of applications, including energy conversion and storage. This paper presents a systematic discussion of current developments in Ti_3_C_2_ MXenes/MOF composites for photocatalytic reduction of CO_2_, and production of hydrogen through water splitting. Initially, the overview and characteristics of MXenes and MOFs are independently discussed and then a detailed investigation of efficiency enhancement is examined. Different strategies such as engineering aspects, construction of binary and ternary composites and their efficiency enhancement mechanism are deliberated. Finally, different strategies to explore further in various other applications are suggested. Although Ti_3_C_2_ MXenes/MOF composites have not yet been thoroughly investigated, they are potential photocatalysts for the production of solar fuel and ought to be looked into further for a range of applications.

## Graphical Abstract

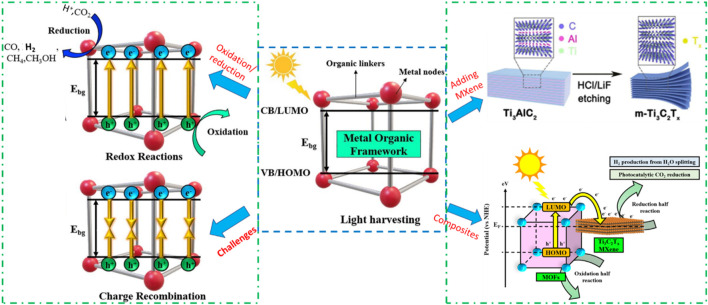

## 1 Introduction

The risks associated with climate change and global warming are becoming worse due to the rising emissions of greenhouse gases, such as CO_2_. Sustainable climate action and renewable energy should complement or replace fossil fuels to reduce this impact (Tahir, 2024). Among the different approaches, the reutilization of greenhouse gas CO_2_ and its conversion to valuable clean products, such as the production of hydrogen as a clean energy source, is a promising pathway to contribute in minimizing the effect of global warming (Jiang et al., 2023; Jiang et al., 2024). Among the available technologies, photocatalysis is one developing technology that can realize this ambition; it has gained popularity as a green technology since it can potentially use renewable sun energy. To achieve this goal, various semiconductor materials are under exploration, however, they have lower efficiency and are unable to fully utilize solar energy (Zhang et al., 2021; Suman, 2018). Addressing substantial overpotential and sluggish kinetics is imperative to enhance the speed of photocatalytic CO_2_ reduction and hydrogen production by applying efficient catalysts. While precious metals exhibit high catalytic activity, their prohibitive cost and limited availability hinder their utilization in large-scale photocatalysis technology. Consequently, photocatalysis requires advancing affordable, non-precious metal-based materials with high catalytic activity and product selectivity (Xie et al., 2018; Liu M. et al., 2020; Zong et al., 2021).

Recently, researchers have given more of their attention to the use of two dimensional (2D) materials which composited of transition metal and carbide materials in areas of catalysis, energy storage and conversion. Several reports unveiled the potential advantages of 2D MXenes layered structure to couple with other semiconductors as co-catalysts and found promising to enhance photocatalytic hydrogen generation and CO_2_ conversion. These materials are considered as a viable alternative to noble-metal catalysts (Qin et al., 2021; Sharma S. K. et al., 2022). Because of their varied chemical composition, MXenes showcase a wide range of intriguing mechanical, electrical, magnetic, and electrochemical characteristics (Han et al., 2019). MXenes due to their 2D layered structure can be combined with several other materials to develop layered configurations which can be beneficial for several applications. Among the MXenes, titanium carbide (Ti_3_C_2_) is a promising material due to its numerous benefits such as increasing catalytic activity and stability, ease of preparation and the inclusion of useful functional groups over the surface. However, MXenes alone have lower efficiency due to strong conductive characteristics, therefore, they can be widely explored as cocatalysts with other semiconductor materials. Thus, MXenes-based composites, sparked considerable interest for their substantial potential in numerous applications, particularly in the realm of photocatalytic CO_2_ reduction and hydrogen generation (Han et al., 2020; Gao et al., 2015).

Metal-organic frameworks (MOFs) have been the subject of an increased investigation for photocatalytic uses in the recent years. Their noteworthy properties, such as their large specific surface area, varied crystalline structures, changeable bandgap, and flexible chemistry and usefulness, are what have sparked this interest (Liu et al., 2022; Lin et al., 2014). However, MOFs’ intrinsic crystalline structure creates structural imperfections such as limited electrical conductivity and electron-hole recombination centres. Ultimately, these limitations limit their efficiency in the process of photocatalysis (Liu S. et al., 2020; Reddy et al., 2020). To overcome the issues, a number of initiatives have been started, including the incorporation of additional semiconductors, naturally occurring nanoparticles, and precious metal nanoparticles into MOFs (Reddy et al., 2021; Wang et al., 2020). When used in conjunction with MOFs, Ti_3_C_2_ MXenes are permitted materials. Charge transport efficiency substantially impacts the efficiency in the production of H_2_ through hydrogen evolution reaction (HER) and CO_2_ reduction to various useful chemicals and products. Thus, to resolve the lower MOF conductivity for the transport of charges, it is necessary to use a highly conductive nature with an ability to trap and transport photoinduced charge carriers. Ti_3_C_2_ MXenes exhibit robust electrical conductivity and exceptional stability. Significantly, Ti_3_C_2_ MXene’s higher conductivity can enhance charge carrier transport efficiency, enabling precise adjustments to catalytic performance in multicomponent catalyst systems such as MXenes/MOF (Wu et al., 2019; Sharma et al., 2022b). Few studies have explored using MXenes-based metal-organic framework (MOF) composites in photocatalytic CO_2_ reduction and primarily focusing on photocatalytic water splitting.

Herein, recent advances in titanium carbide (Ti_3_C_2_) MXenes and MOF-based composites for photocatalytic CO_2_ reduction and water splitting to produce hydrogen has been disclosed. Initially, an overview and characteristics of utilizing both materials have been discussed. The latest advancements in utilizing MXenes and MOFs composites such as binary and ternary heterojunction formations for applications such as photocatalytic hydrogen (H_2_) production, and carbon dioxide (CO_2_) reduction are demonstrated. The discussion predominantly delves into the characteristics and performance of previously documented nanocomposites and nanohybrids featuring Ti_3_C_2_ MXene-based MOFs. Finally, this review outlines existing challenges and potential avenues for future research in Ti_3_C_2_ MXene-based MOF composites. Although not much research has been done on Ti_3_C_2_-MOF composites, it is obvious that they are potential photocatalysts for the production of solar fuel and that more research should be done on them for a range of uses.

## 2 Overview of MOFs and titanium carbide MXenes

### 2.1 Overview of MOFs

Metal-organic frameworks (MOFs) are porous materials characterized by a high specific surface area and a three-dimensional structure. The fundamental units of MOFs are created by joining metal clusters with organic ligands to create a three-dimensional organized network (Karthik et al., 2017). In comparison to other semiconductors, these materials, significantly, have a greater specific surface area due to their increased porosity, which is a result of their geometrically well-defined framework structure (Ren et al., 2024). Although these materials had been acknowledged earlier, significant attention was drawn to MOFs in the late 1990s due to the work reported by Omar Yaghi and his colleagues. They were able to synthesize extraordinarily rigidly synchronized networks with organic linkers and metal ions known as MOF-5 (Li et al., 1999; Li D. et al., 2019). Due to the distinctive and exceptional qualities inherent in MOFs, there has been a notable surge among researchers focused on developing new materials and exploring their applications. MOFs exhibit significant potential across diverse fields, including gas sorption and separation, luminescence, catalysis, proton conductivity, and sensor development. Their extremely configurable features, higher crystallinity, long-lasting porosity, incredibly huge surface area, compelling and extensive permutations, and highly changeable framework topology are all responsible for this (Li D. et al., 2019; Zhou and Kitagawa, 2014; Li et al., 2009; Corma et al., 2010; Cui et al., 2012).

**FIGURE 1:**
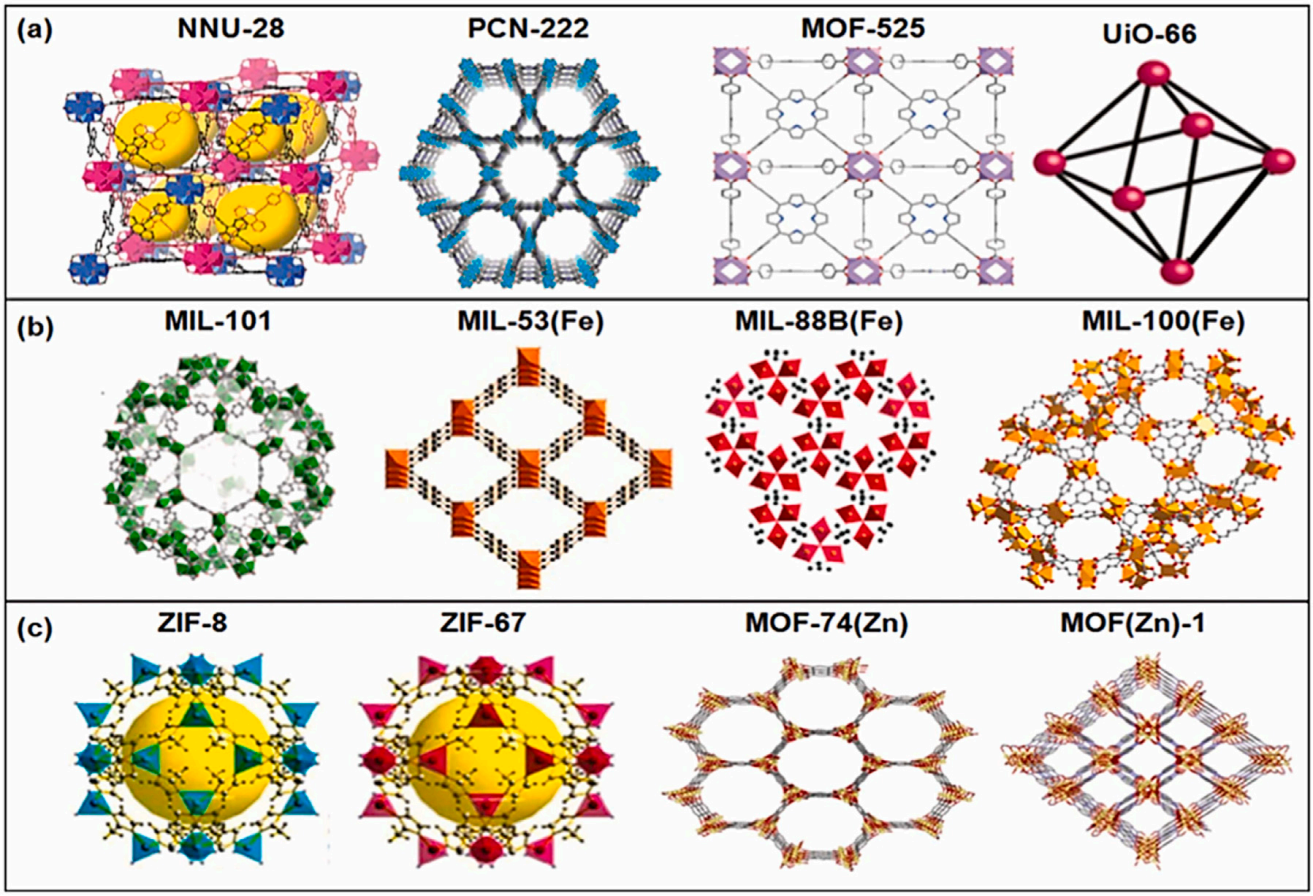
**(A–C)** Developments of MOFs and their various structures. Reproduced with permission from (Tahir et al., 2023a). Copyright 2023 Elsevier.

In addition to MOF as a pure material, they can be converted to several metal oxides and porous materials with a larger surface area and abundance of surface-active sites. In the last 10 years, MOF research has drawn a lot of interest from the chemistry and materials science communities. Heterogeneous catalysis was one of the many uses of MOFs that was first investigated in 1994 (Fujita et al., 1994). It continues to capture the attention of researchers owing to its chemical adaptability, custom-designed pore structures, and expansive, easily accessible internal surface areas. There are four primary categories of MOF materials based on their synthesis: 1) Zeolitic imidazolate frameworks (ZIFs), 2) Matériaux de l′Institut Lavoisier (MILs), 3) Universitet I Oslo (UIO), and 4) Isoreticular Metal-Organic Frameworks (IRMOFs) (Zhao et al., 2022). MOFs have metal clusters in the parent MOFs, primarily transitional ones like Ti, Zr, Fe, and Zn. Figures 1A–C shows the recent developments in various MOFs and their structures and uses in various applications (Tahir et al., 2023a).

MOFs are distinguished from all other solids by their exceptional qualities of high specific surface area and porosity, which are a result of their structural and tunability traits. Because of their strong physical adsorption capabilities, MOFs, a porous material with a large surface area, are thought to be the best adsorbents for gas storage and separation, primarily of CO_2_ and H_2_. However, by using analogies with other related MOF structures and the relative dimensions of the ligands, it is possible to anticipate, change, and control the pore shape/size, and dimensions (Li et al., 2011).

### 2.2 Structure and properties of MOFs

MOF structure consists of organic linkers that connect the secondary building blocks to form regularly spaced porous structures, which can be either clusters or metal ions (Jiao et al., 2019). The vast diversity of MOFs arises from the numerous possible combinations of these structural elements. The multifunctionality of MOFs is rooted in their diverse crystalline forms. Secondary building units (SBUs) in MOFs which can either be metals or metal clusters are coupled with organic linkers, primarily carboxylic acids or nitrogen-containing ligands, to form MOFs.

Unlike other porous materials like carbons and zeolites, MOFs possess a unique tunability that sets them apart. The configuration of MOFs is defined by the size, shape, and arrangement of organic linkers relative to the SBUs (Tahir et al., 2021a). Through careful selection of SBUs and connectors, MOFs can be finely adjusted, enabling customization of structure, pore size, shape and functionality to meet required application requirements. Theoretically, MOF morphology/structures can be predicted by considering the organic linker-building components and metals involved. It is worth noting that a database containing over 20,000 recorded MOF forms has recently become available, and further are under exploration (Jiao et al., 2019; Furukawa et al., 2013). MOFs having specific shapes and structures facilitate higher light penetration and reactant attachment. In MOF having active sites can help organic bonds with functional groups such as amine and pyridyl to make it possible to identify particular tiny molecules.

It is possible to add functional groups to SBUs or organic linkers after synthesis that are not suitable for MOF synthesis. The structure and type of MOF and their characteristics such as active sites, surface area and functional groups all depend on the type of metal ions/clusters and organic linkers. MOFs have distinct magnetic, electrical, and optical properties that can be precisely altered to achieve specific objectives. Moreover, MOFs’ pore space can hold a variety of functional hosts with multitasking capabilities. MOFs are resistant to a wide range of species, including organic dyes, nanoparticles, polyoxometalates, single metal atoms, metal complexes, tiny enzymes and polymers (Jiao et al., 2019; Perry Iv et al., 2009; Tranchemontagne et al., 2009; Lu et al., 2014; Deng et al., 2012). The spectrum of possible uses within a single MOF is greatly expanded upon the introduction of a guest species (Jiao et al., 2019). In the past 20 years, numerous novel compounds have been discovered, however, it has been observed that MOFs have poor stability with acid/base, lower thermal stability, and unacceptable mechanical properties. Therefore, ensuring the resilience of MOFs is crucial for many real-world applications. Over the last few years, several initiatives have been launched, resulting in significant progress in addressing this challenge (Burtch et al., 2014; Howarth et al., 2016; Bosch et al., 2014). Numerous MOFs with water stability have been extensively investigated such as the MIL-101 series based on chromium being explored to date (Férey et al., 2005), MAFs (metal isolate frameworks) (Huang et al., 2006), ZIFs (zeolitic imidazolate frameworks) (Park et al., 2006), pyrazole-based MOFs (Colombo et al., 2011), the aluminium-based carboxylates (Loiseau et al., 2004) and the zirconium-based carboxylates (Furukawa et al., 2014; Cavka et al., 2008; Feng et al., 2012). To enhance MOFs’ resistance to water and moisture, recent efforts have concentrated on imparting hydrophobic surfaces or interfaces to them. For example, Nguyen and Cohen employed a medium with extended alkyl groups as an effective approach to safeguard moisture-sensitive MOFs (Nguyen and Cohen, 2010). Due to their super-hydrophobic nature, fluorinated MOFs (FMOFs) were reported to have good water stability (Yang et al., 2011). On the other hand, for gas phase reaction that can occur at high temperatures such as dry reforming of methane, thermal stability is also very important for their practical applications. Controlling thermal stability frequently means to select a suitable and more linkers with each metal node with their stronger connections. The metal ions with higher valence state such as Ti^4+^, Zr^4+^, Ln^3+^, and Al^3+^ are frequently utilized to produce MOFs with their higher thermal stability. While most MOFs are stable between 350 and 400°C, a few, such as MIL-53 (Loiseau et al., 2004) and UiO-66 (Cavka et al., 2008), are stable over 500°C. Because thermal stability offers a consistent indicator of resistance to other stressors, it is frequently the only type of stability tested for novel MOFs (Jiao et al., 2019).

Most MOFs under explorations are microporous which exhibits an excellent gas adsorption property, particularly for gases like hydrogen and carbon dioxide, with pore diameters typically around 2 nm. Large surface areas and substantial micropore volumes are desirable for many applications, yet such tiny holes cannot support enormous particles or molecular processes. Their limited ability to facilitate rapid mass diffusion and transfer makes them less useful for medication administration, storage, separation, and catalysis (Jiao et al., 2019). For some more contemporary applications, such as drug delivery and catalysis, mesoporous MOFs with their size in the range of 2 to 50 nm are most favoured (Furukawa et al., 2013; Xuan et al., 2012).

Large pore-size MOFs can be created and modified using a variety of efficient techniques. Lengthening ligands is an obvious and practical method to do this (Eddaoudi et al., 2002). The record for the largest pores in MOFs is now held by IR-MOF-74-XI, which has an amazing pore diameter (Feng et al., 2012). The functionalities of MOFs depend on both the pore diameter and the Langmuir and Brunauer-Emmett-Teller (BET) surface areas (Senkovska and Kaskel, 2014). The surface area of NU-110 is the highest of any known material, at 7,140 m^2^ g^−1^ (Farha et al., 2012). Based on calculations, the maximum possible surface area of MOFs can be reached to 14,600 m^2^ g^−1^ (Farha et al., 2012), however, determining this number experimentally remains challenging (Jiao et al., 2019).

Despite having a relatively high specific surface area, the majority of MOFs that have been described have poorer electrical conductivity. MOF’s low conductivity is caused by its tight charge localization and low electron density, whereas charge carriers are trapped on the lattice sites. The conductivity of MOFs can be enhanced by developing new strategies for their synthesis with mandatory characteristics such as the band transport within their metallic crystal structure. In a recent review paper by Ren and co-authors, the conductive characteristics of various MOFs have been summarized. For example, Cu [Ni(pdt)_2_] MOF depicted conductivity of 1 × 10^−4^ S cm^−1^ and Co-HAB has an electrical conductivity of 1.57 S cm^−1^. Similarly, many other MOFs have an electrical conductivity of 1.3 × 10^−7^ S cm^−1^ for NU-1,000 and 1580 S cm^−1^ for Cu-BHT. The MOF conductivity can be increased through variety of ways with respect to their synthesis and surface modification (Ren et al., 2021). Additionally, MOFs containing organic linkers may be able to absorb light and become functionalized by adding other groups, such as amino groups. The conductive characteristics of MOFs can also be improved by their contact with highly conductive materials with matched Fermi levels. In this perspective, MOF combined with MXenes and in particular titanium carbide (Ti_3_C_2_) MXenes can be beneficial to enhance photocatalytic efficiency. The detailed properties and characteristics of MXenes are discussed in the following sections.

### 2.3 Overview of Ti_3_C_2_ MXenes

MXenes are a type of 2D transition metal carbide that can be likened to graphene. M_n+1_X_n_T_x_ (n = 1 to 3) is the general MXenes formula, however, M, X and T can be varied depending on the types of MXenes and metals involved. So far, several metals (M) MXenes are used to produce different type of MXenes such as Ti, Sc, V, Zr, Cr, Ta, Mo, Nb, Hf, and Mn. The T in the MXenes represent the surface termination element, which can be oxygen (-O), hydroxyl (-OH), or fluorine (-F) (Hong W. et al., 2020; Gogotsi and Anasori, 2019). The recent discovery of novel 2D transition metal carbides, nitrides, and carbon nitrides—referred to as MXenes—has reignited interest in investigating cutting-edge ideas and their possible uses. Surface terminations arise as a result of exposure to the environment, affecting MXenes’ chemically active exterior layer. MXenes can be classified into mono-M, double-M, or solid-solution M elements based on their atomic lattices and composition (Jiang et al., 2020; De et al., 2022).

**FIGURE 2:**
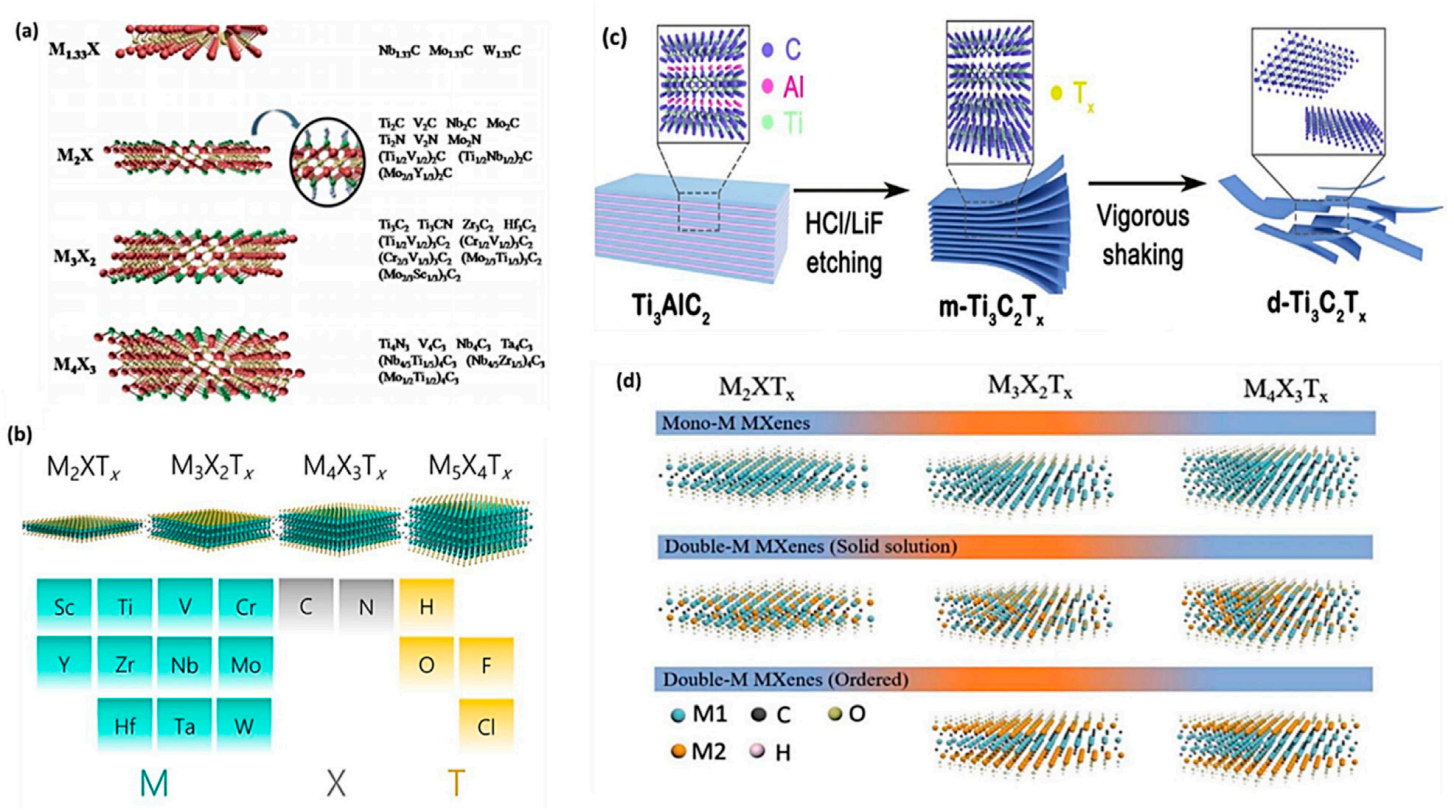
**(A,B)** Illustration of structural categories of 2D MXenes, **(C,D)** Structural classification of MXenes. Reproduced with permission from (Tahir et al., 2023b). Copyright 2023 Elsevier.

Figures 2A–D shows different combinations of elements to produce MXenes with their different structures. The crystal structure of MXenes is typically hexagonal closed-packed, but different MXenes exhibit distinct sequences of M atoms. For instance, the face centre cubic sequences of M_3_X_2_ and M_4_X_3_ are different from the ABABAB pattern of M_2_X’s hexagonal closed-packed structure (ABCABC). Different MAX ternary carbides and nitrides are utilized in the MXenes class to create a variety of MXenes, such as Ti_2_C, Ti_3_CN, Cr_2_TiC_2_, MO_2_C, V_2_C, Ti_3_C_2_, Zr_3_C_2_, Ti_4_N_3_, and Mo_2_ScC_2_ (De et al., 2022; Anasori et al., 2017). Among several MXenes, Ti_3_C_2_, which is etched from the hexagonal compressed MAX parent material (Ti_3_AlC_2_) by weak Ti–Al bond breaking, is recognized as a hotspot for MXenes-based photocatalysts.

### 2.4 Structures of MXenes

**FIGURE 3:**
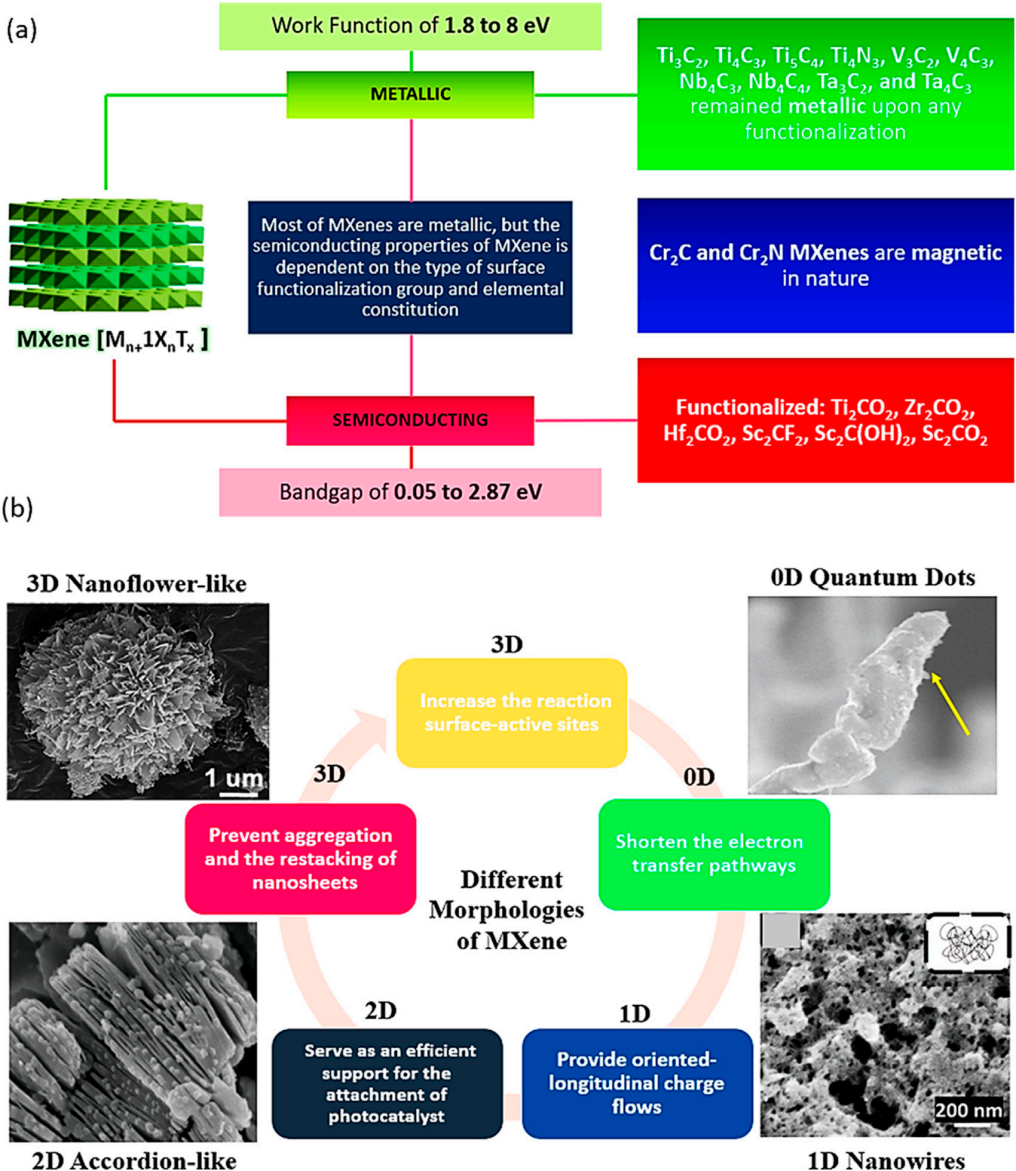
**(A)** Characteristics of different types of materials, **(B)** Morphological classification of MXenes.

MXenes are composed of earth-abundant and non-toxic elements, forming 2D substances. Furthermore, when compared to other 2D materials like graphene, MXenes are thought to be superior due to their hydrophilic nature and metal-like electric conductivity. These features are as follows: i) effective metallic hydroxide sites; ii) complex surface chemistry; iii) enhanced electronic conductivity; iv) remarkable chemical durability; v) resistance to corrosion and vi) modifiable optoelectronic properties that can be achieved by varying the particle size, layer spacing, and layer quantity (Sharma et al., 2022b; Liu and Dong, 2021; Lim et al., 2020). In the M_n+1_X_n_T_x_ formula, representing termination count, the robust metallic connections between M and A are disengaged, giving way to less formidable links such as (-O, -F, or -OH, for instance. Three distinct MXenes kinds—M_2_X, M_3_X_2_, and M_4_X_3_—appear after etching as presented in Figure 2. They are all composed of the same hexagonal closed-pack crystal structure, where the MX octahedrons are surrounded by X atoms. Transition metal honeycomb lattices arranged in a hexagonal pattern are present on the upper layer of the M_2_X phase. Regarding the M_3_X_2_ and M_4_X_3_ phases, they have out-of-plane structures where other atoms occupy the middle region while transition metals are located on the outer layer (Sharma et al., 2022b; Khazaei et al., 2017). The M_2_X phase consists of three-layer sheets with X (either C or N) positioned between 2 M layers. Within the early transition metal strata is where X is located. In the M_2_X phase, there are two hollow spaces between the transition metal interlayers, each containing one “X” atom or none at all (Sharma et al., 2022b; Li and Wu, 2019). The M_3_C_2_ and M_4_C_3_ phases have a face-centred cubic stacking structure. The attributes of MXenes include a strong electrical and thermal conductivity, a programmable band gap, and a high Young modulus. Graphene and most other 2D materials are not like MXenes due to their hydrophilic surfaces and strong metallic conductivities (Alhabeb et al., 2017; Persson et al., 2018; Huang et al., 2018). Ultimately, their properties and performances can be altered by alterations in i) composition, ii) surface functionalization and iii) structure/morphology (Anasori et al., 2017; Halim et al., 2018; Kong et al., 2018; Kayali et al., 2018; Papadopoulou et al., 2020; Ronchi et al., 2019). The different properties of Ti_3_C_2_ MXenes are summarized in Figure 3A.

Jiang et al. (2020) in their work indicated that MXenes, which are not terminated, typically exhibit metallic properties characterized by a substantial density of states (DOS) in the vicinity of the Fermi region. This is explained by an exterior layer made up of metallic transition elements. The p-electrons of the X atoms contribute to energy bands that are situated between −3 and −5 eV below the Fermi surface and d-electrons of the transition metals that surround the Fermi surface mostly affect the DOS (Enyashin and Ivanovskii, 2013; Khazaei et al., 2012). The electrical characteristics of MXenes are said to be more influenced by the outside transition metal layers than by the interior transition metal layers (Ivanovskii and Enyashin, 2013; Anasori et al., 2015; Dong et al., 2017). Hence, surface terminations linked to transition metal atoms in the outer layer possess the capability to notably modify electrical properties, including band structures. One or two electrons from the outer transition metal layers are absorbed by the electronegative termination, reducing the density of states (DOS) below the Fermi surface and creating a new energy band. Both -OH and -F groups exert similar effects on the electrical arrangement of MXenes since they can only accept one electron each. However, an O-group can accept two electrons, resulting in a more significant impact. In MXenes, surface functional groups also influence thermal and electronic transport. For F-terminated MXenes, electronic transmission is excellent, whereas surface functionalization with O atoms significantly diminishes electronic communication (Berdiyorov, 2015).

Until yet, the electrical properties of terminated MXenes have been identified by tests or anticipated theoretically; these properties range from extremely conductive metallic states (Ying et al., 2017; Dillon et al., 2016; Urbankowski et al., 2017) and from semiconducting to highly insulating topological states (Si et al., 2016). Stoichiometry tailoring can modify the energy structure of MXenes, except for surface terminations (Wong et al., 2018), doping (Balcı et al., 2017), an external electric field (Balcı et al., 2018), crystal lattice symmetry (Hong et al., 2016), and stresses (Lee et al., 2014). Over the past few years, MXenes have demonstrated several fascinating optical characteristics. These demonstrate effective photothermal conversion, plasmonic behaviour, and optical transparency (Papadopoulou et al., 2020). The diverse ways in which MXenes interact with light have greatly influenced study. Again, a material’s optical characteristics are largely determined by its surface terminations (Papadopoulou et al., 2020; Chaudhuri et al., 2019). The optical characteristics of MXenes can be adjusted by modifying the intrinsic properties of transition metals (Halim et al., 2019). Ti_3_C_2_T_x_’s optical non-linearity was claimed to be achieved by using excitation sources at several wavelengths, including 800, 1,064, 1,550, and 1800 nm (Jiang et al., 2018). These compounds are highly suitable for photocatalytic applications because of their functional groups (such as -OH and -O groups) and wide surface area (Jiang et al., 2020; Jeon et al., 2020; Shi et al., 2021).

Since their discovery in 2011, a novel and expanding class of transition-metal carbides, nitrides, and carbonitrides known as MXenes has emerged. MXenes represent a next-generation nanomaterial for investigating sustainable energy resources, particularly in catalysis for energy and environmental technologies, owing to their intriguing electrical and structural properties. Figure 3B illustrates various MXenes topologies, including 0D, 2D, and 3D structures, which have been explored for a range of applications over time.

Zero-dimensional (0D) Ti_3_C_2_ quantum dots offer several advantages over their two-dimensional (2D) counterparts. The quantum confinement effect results in a wider band gap, a higher negative Fermi level, an increased number of active edge sites, and enhanced dispersibility for Ti_3_C_2_ quantum dots compared to Ti_3_C_2_ sheets. Similar to 2D Ti_3_C_2_ sheets, Ti_3_C_2_ quantum dots can serve as electron acceptors to facilitate carrier migration (Xu et al., 2023).

### 2.5 Properties of MXenes

MXenes are known for having higher electrical conductivities than multi-layered graphene, surpassing other multi-layered materials such as carbon nanotubes and reduced graphene oxide (Bansal et al., 2024). The conductivity of MXenes is dependent on the presence of surface functional groups such as -OH, -F, and -Cl. To enhance electrical conductivity, surface -OH groups can be substituted with -F or -Cl groups. Additionally, conductivity can be boosted by doping MXenes with elements like carbon or nitrogen or by introducing surface imperfections such as vacancies or dislocations. Intercalation serves as another method to increase conductivity. MXenes fabricated with shorter etching intervals and lower HF concentration levels tend to exhibit larger lateral dimensions and fewer defects, resulting in higher electronic conductivities—up to five times higher in larger flake sizes compared to smaller ones. Thermal and/or alkaline treatments effectively enhance the electrical properties of MXenes, leading to conductivity increases by two orders of magnitude. This improvement is dependent on the functional groups, particularly -F and also it changes with the addition or removal of intercalated molecules (Alli et al., 2024).

While MXenes show promise for a variety of applications, their stability presents a significant challenge for long-term use in real-world settings. Atomic-scale flaws and storage conditions lead to rapid oxidation, which alters their microstructure and degrades their electrochemical properties. Additionally, MXenes tend to react with trace amounts of oxygen, even in controlled environments. This instability, which can occur at room temperature or below, means that the preparation and storage methods of MXenes critically influence their final characteristics.

## 3 Applications of Ti_3_C_2_-based MOF composites for CO_2_ reduction

### 3.1 MOF-based photocatalytic CO_2_ reduction

In photocatalytic CO_2_ reduction applications, the performance of a semiconductor is dependent on the band gap energy, specific surface area, electrical conductivity, light harvesting efficiency and charge transfer ability (Bao et al., 2024; Zhou et al., 2024). Metal-organic frameworks (MOFs), because of their excellent CO_2_ adsorption properties, large surface areas, flexible structures and tunable optical properties, have recently gained attention as promising photocatalysts for CO_2_ reduction. Since photocatalysis uses solely naturally occurring solar energy as the reaction system energy input, it is a relatively new and sustainable method of inducing catalysis. MOFs can provide a high surface area with a porous structure to maximize the attachment of reactants and also to enable the use of UV and visible light irradiations.

**FIGURE 4:**
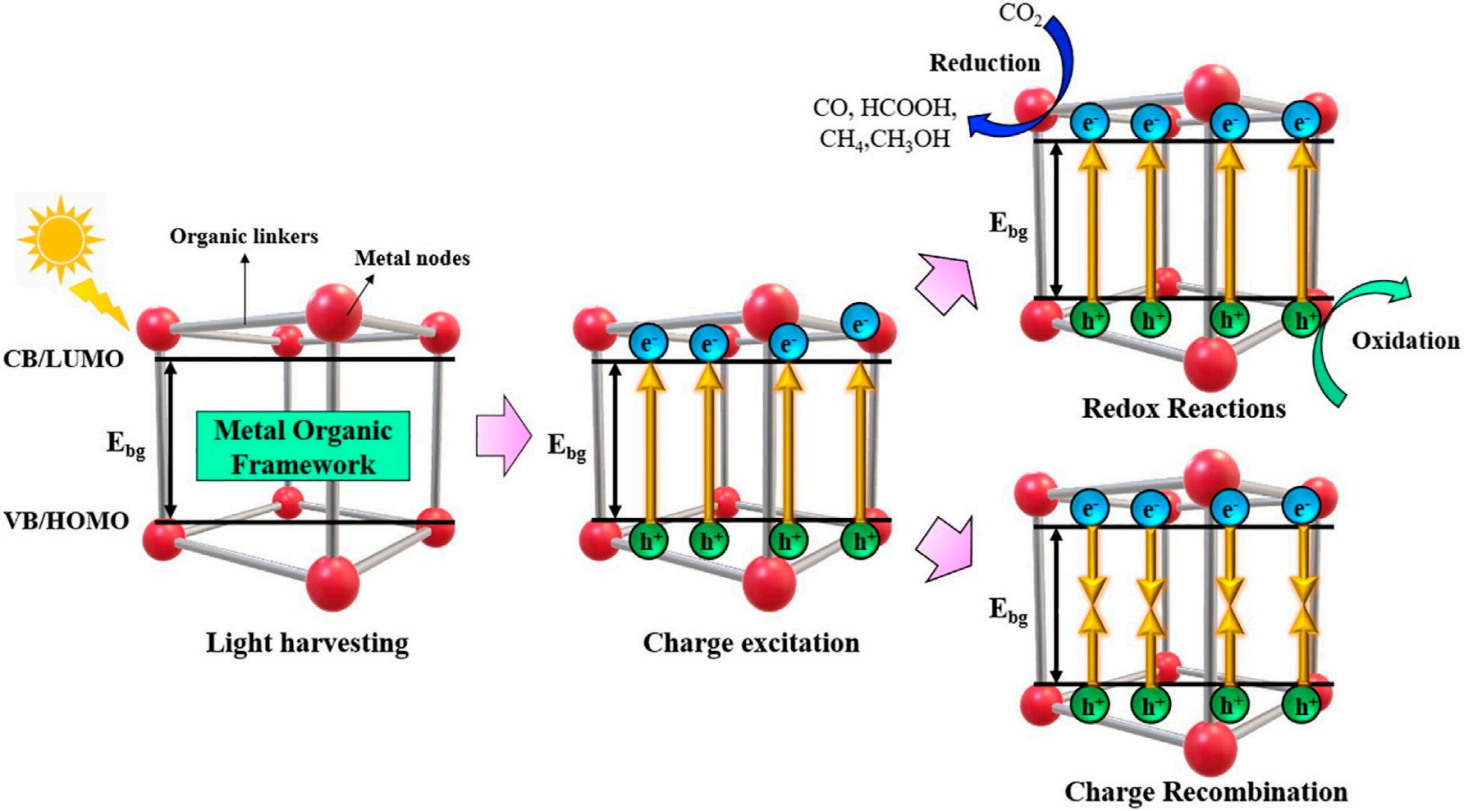
Schematic showing the general process for producing and reducing CO_2_ through photocatalysis. Reproduced with Permission from (Fan and Tahir, 2022). Copyright 2022 Elsevier.

Figure 4 illustrates the four essential phases that make up the photocatalysis pathway. These are the occurrence of a redox reaction, light harvesting, charge excitation, and charge carrier recombination (Sherryna et al., 2021). The photo-responsive materials are initially subjected to light radiation. There are two kinds of charge transfer in MOFs: band-like transport and metal-ligand bond transport. In the case of metal-ligand transport, the interaction between ligand π and metal d-orbital builds a donor-bridge-acceptor path, which in compounds with mixed valence can encourage electron transport. The conductivity can be enhanced by this kind of electron transfer up to a magnitude of 10–10^2^ S cm^−1^. An electrical conductivity of 1580 S cm^-1^ at room temperature can be attained by the use of band-like transport (Ren et al., 2021).

The mechanism of CO_2_ reduction to various products is further shown by Equation 1–5 (Bao et al., 2024). The reduction of CO_2_ to CO_2_^−^ radical is illustrated in Equation 1, however, this reaction has a high redox potential, thus it is considered unrealistic (Costentin et al., 2013). Furthermore, a photocatalyst cannot provide enough potential to move even one electron to a CO_2_ molecule (Habisreutinger et al., 2013). Reactions with low redox potentials can occur in a variety of materials (Barton Cole et al., 2010), due to their characteristics as proton-assisted multi-electron reactions. Equation 2–5 illustrate that using photocatalysis, CO_2_ can be converted to a variety of chemicals and useful products including CO, HCOOH, CH_4_, CH_3_OH and others. The quantity of electrons provided to the CO_2_ molecule determines the product selectivity. For example, it often favours the formation of CO and HCOOH because the process requires just two electrons to start the reaction. However, for the formation of CH_3_OH and CH_4_ an additional 6 and 8 protons and electrons, respectively, are needed (Sherryna and Tahir, 2022a; Huang et al., 2019).

$${\text{CO}}_{2}+e^{-}\;\to \;{\text{CO}}_{2}^{-}\;\left(-1.90\text{ eV}\right)$$ (1)

$${\text{CO}}_{2}+2H^{+}+2e^{-}\;\to \text{ CO}+H_{2}O\;\left(-0.52\text{ eV}\right)$$ (2)

$${\text{CO}}_{2}+2H^{+}+2e^{-}\;\to \text{ HCOOH }\left(-0.61\text{ eV}\right)$$ (3)

$${\text{CO}}_{2}+6H^{+}+6e^{-}\;\to \;{\text{CH}}_{3}\text{OH}+H_{2}O\;\left(-0.38\text{ eV}\right)$$ (4)

$${\text{CO}}_{2}+8H^{+}+8e^{-}\;\to \;{\text{CH}}_{4}\left(g\right)+2H_{2}O\;\left(-0.24\text{ eV}\right)$$ (5)

In photocatalysis, after light harvesting is successful, electrons are stimulated to a high enough energy to cross the E_bg_ and move from the highest occupied molecular orbital (VB) (HOMO) to the valence band (VB), also known as the lowest unoccupied molecular orbital (LUMO). Alternatively, the holes created by the photons are subsequently retained in the VB or HOMO (Fan and Tahir, 2022). After the electron/hole pairs are created during the charge excitation step, two things can happen: either the charge carriers recombine unfavourably, resulting in energy loss, or the electron/hole pairs are employed favourably for redox reactions. The CO_2_ reduction occurs over CB or LUMO with the involvement of photogenerated electrons and protons to convert it into various products (Tahir et al.). Recombination can take place in two different ways: either on the surface of the material, where an electron/hole has the potential to recombine or they can even recombine in the bulk (Khan and Tahir, 2019).

MOFs have a higher surface area which enables to provide more active sites for the attachment of reactants. In photocatalytic applications, higher visible light absorbance and superior separation of photoinduced charge carriers are essential to maximize photocatalytic efficiency. The light absorbance of MOFs from the UV to visible can be obtained by changing the colour. These materials, however, have poor electrical conductivity, and become a major obstacle in photocatalytic applications (Ren et al., 2021). Hence, it is crucial to prevent recombination by employing various techniques such as sensitizing using cocatalysts and heterojunction formation. These methods can trap electrons and induce spatial separation, thereby prolonging the lifespan of photo-generated electron/hole pairs.

### 3.2 Ti_3_C_2_/MOF composites

In the photocatalytic CO_2_ reduction process over the MOF and other semiconductor materials, one of the biggest challenges is the lower efficiency and product selectivity. Pure MOFs exhibit photocatalytic activity for the generation of solar fuel but with lower efficiency (Li N. et al., 2019; Liao et al., 2018) and the rapid recombination of charge carriers is the reason for lower productivity. Ti_3_C_2_ MXenes are thus employed to regulate the photoactivity of MOF. Ti_3_C_2_ MXenes can function as co-catalysts to establish Schottky junctions with other photo-active materials to efficiently capture photogenerated electrons because of their metallic characteristics. It has been suggested to combine MOF with more conductive materials and create binary composites in order to overcome the barrier causing low photocatalytic activity and efficiency.

Numerous investigations on 2D layered Ti_3_C_2_ MXenes and their composites such as Ti_2_C_3_/MOF and their application in photocatalytic solar fuels production have been carried out in the past few years (Li et al., 2022). MOF composites based on Ti_3_C_2_ MXene offer some beneficial synergies. First off, MXenes can be hosted by MOFs, which have a high porosity and surface area and can stop MXenes layers from aggregating and restacking. Because of the synergistic effects between the functions of MOFs and the surface terminal groups of MXenes, the composite is also able to demonstrate increased stability (Najam et al., 2022). Furthermore, the MXenes and MOF create an intrinsic electric field which is associated to Schottky junction, which facilitates the effective separation, quick mobility, and transfer of charge carriers (Yu et al., 2021). Ti_3_C_2_ MXenes-based MOF heterojunctions can serve as appealing photocatalysts for water-splitting-based H_2_ production and CO_2_ reduction. Nevertheless, the composite for the manufacture of solar fuel has been the subject of very few studies.

**FIGURE 5:**
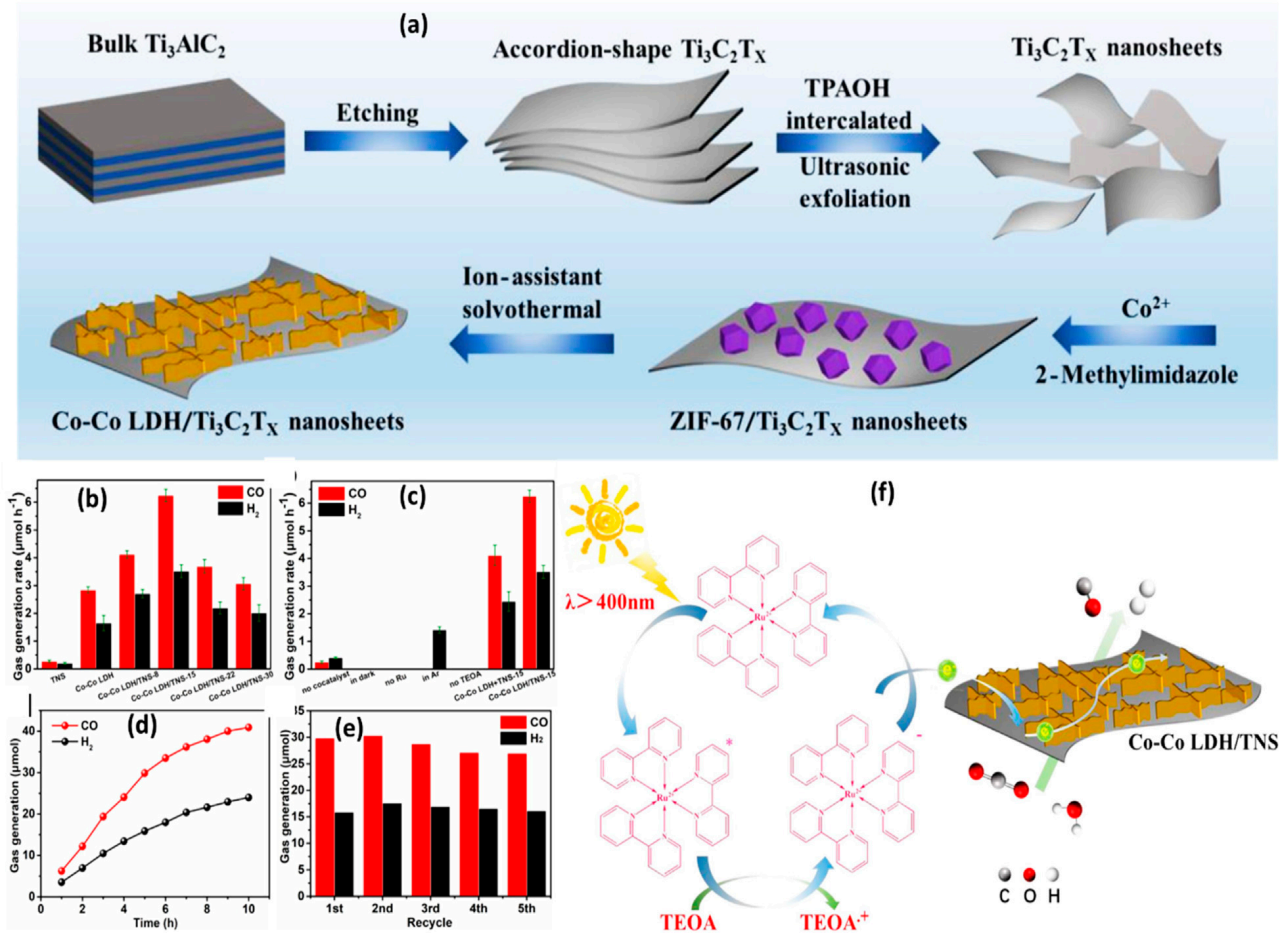
**(A)** A schematic diagram illustrating the process of creating Co-Co LDH/Ti_3_C_2_T_x_ nanosheets using MOF; **(B–E)** photocatalytic activity and stability for the CO_2_ reduction process over Co-Co LDH/Ti_3_C_2_T; **(F)** The mechanism of CO_2_ reduction over the nanocomposite using TEOA as a sacrificial agent and [Ru (bpy)_3_]Cl_2_ photosensitizer. Reproduced with permission from (Chen et al., 2020). Copyright 2020 Elsevier.

The use of binary Ti_3_C_2_ composite with Co-Co LDH nanosheets produced from MOF for CO_2_ reduction with the involvement of [Ru (bpy)_3_]Cl_2_ sensitizer under visible light is shown in Figure 5A. Ultrasonic exfoliation was used to produce Ti_3_C_2_T_x_ nanosheets after the bulk Ti_3_AlC_2_ MAX was etched to remove the Al layers (TNS). Next, ZIF-67 was grown *in situ* on the Ti_3_C_2_T_x_ nanosheets. After ZIF-67 was successfully loaded, the nanocomposite underwent solvothermal treatment to produce Co-Co LDH/TNS nanosheets. No CO_2_ reduction activity was observed by photocatalytic in pristine Ti_3_C_2_T_x_ nanosheets. However, as Figures 5B–D illustrates, there was a noticeable increase in the rate of CO generation over MOF-derived Co-Co LDH during the CO_2_ reduction process. As shown in Figure 5E, the nanocomposite also demonstrated outstanding stability, retaining strong photocatalytic activity for up to five cycles. Figure 5F shows the Co-Co LDH/TNS heterojunction to maximize the charge separation efficiency and light absorbance ability of the composite using a photosensitizer. The photosensitizer is activated by visible light irradiation, however, TEOA works as a hole scavenger and electron donors are used to generate the reduced state [Ru (bpy)_3_]Cl_2_. After that, the electrons are transferred to the nanocomposite, where they quickly move to the co-active sites to reduce CO_2_ to CO (Chen et al., 2020).

**FIGURE 6:**
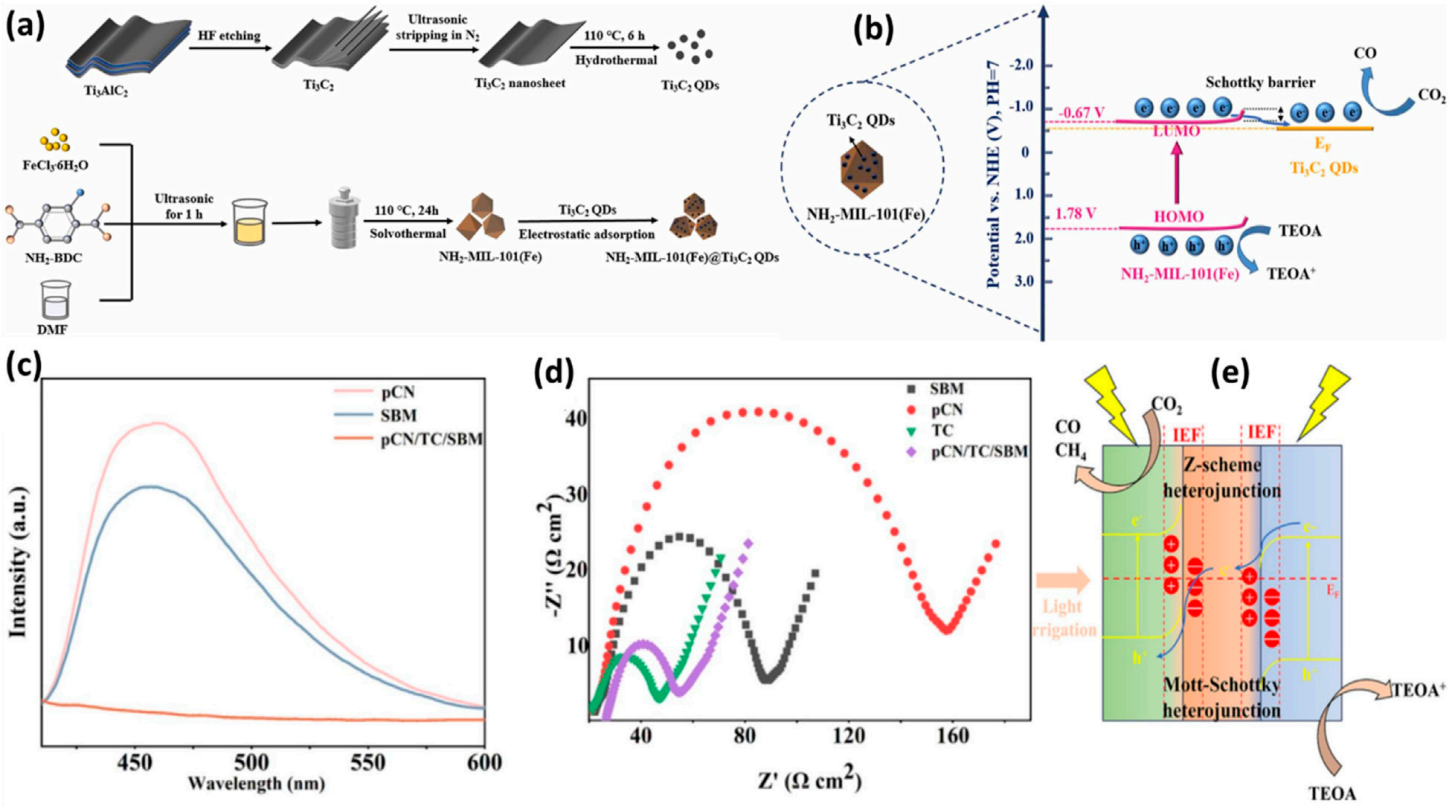
**(A)** Schematic for the synthesis of Ti_3_C_2_ QDs assisted NH_2_-MIL-101 composite, **(B)** Schematic illustration of charge separation over Ti_3_C_2_ QDs/NH_2_-MIL-101 composite. Reproduced with Permission from (Xu et al., 2023). Copyright 2023 Elsevier; **(C,D)** PL and EIS spectra of pCN, SBM, pCN/TC/SBM samples, **(E)** The photocatalytic mechanism of the pCN/TC/SBM. Reproduced with Permission from (Song et al., 2024). Copyright 2024 Elsevier.

The Ti_3_C_3_ MXenes were further investigated with other Fe-based MOFs. Because of their outstanding CO_2_ adsorption capabilities, wide surface areas, high porosities, tuneable optical properties, and flexible architectures, Fe-MOF has recently shown great promise as a photocatalyst for CO_2_ reduction. However, due to their rapid carrier recombination and limited charge transport efficiency, pure Fe-MOF exhibits comparatively low photocatalytic activity. In recent work, Ti_3_C_2_ QDs coupled with NH_2_-MIL (101) were examined for photocatalytic CO_2_ reduction applications (Xu et al., 2023). As seen in Figure 6A, a straightforward electrostatic adsorption method was used to create the T_3_C_2_ QDs on the surface of 3D octahedral NH_2_-MIL-101(Fe). By hydrothermally reacting Ti_3_C_2_ in an N_2_ environment and ultrasonic stripping, Ti_3_C_2_ quantum dots of size of 4–5 nm were produced. Because of the -NH_2_ groups, NH_2_-MIL-101(Fe) has a wide light absorption range from UV to visible light. The hybrid NH_2_-MIL-101(Fe)/Ti_3_C_2_ QD samples exhibit higher light absorption than the NH_2_-MIL101(Fe), and the absorption intensity increases as the Ti_3_C_2_ content increases. For NH_2_-MIL-101(Fe), NMTQ0.5, NMTQ0.75, and NMTQ1.0, the corresponding band gap energy values are 2.45, 2.41, 2.29, and 2.15 eV, were reported. The decrease in charge recombination rate was also observed with QDs loading to Fe-MOF.

As discussed previously, Ti_3_C_2_ QDs have the potential to increase visible light and charge separation efficiency, which was beneficial to increasing CO_2_ reduction efficiency to maximize CO formation during the photocatalysis process. Due to good interface interaction, this binary composite was stable enough to produce CO for consecutive five cycles. Effective charge separation and band modification were responsible for the remarkable CO_2_ photoreduction activity of NH_2_-MIL-101(Fe)/Ti_3_C_2_ QDs, as shown in Figure 6B. Under visible light, NH_2_-MIL-101 (Fe) produces electrons and holes and due to good interface contact photoexcited electrons migrated to Ti_3_C_2_ QDs because of the low Fermi energy level and excellent charge transfer efficiency. The formation of the Schottky barrier prevents electrons from backflowing and recombining with holes, resulting in significantly increased CO_2_ photoreduction efficiency.

In a recent development, a trimetallic Sn/Ti_3_C_2_-supported Bi-MOF was tested for the conversion of CO_2_, and the potential products are discussed in Figure 6 (Song et al., 2024). First, Ti_3_C_2_ MXene was prepared by passing LiF-HCl through Ti_3_AlC_2_ MAX and etching it for 48 h at 60°C. The g-C_3_N_4_-Sn-Bi-MOF exhibited a good interaction, achieved using a straightforward electrostatic self-assembly process.

The photochemical characteristics of the produced catalysts were investigated using UV-visible spectroscopy. The absorption band limits for pCN/SBM and pCN/TC/SBM are observed at wavelengths of 492 nm and 493 nm, respectively. Additionally, TC shows no discernible absorption edge, indicating its nature as a pure electrical conductor without light absorption. The composite photocatalysts, as shown in Figure 6C, exhibit reduced photoluminescence (PL) intensity, suggesting a low recombination rate of photogenerated electron-hole pairs in pCN/TC/SBM. Furthermore, in Figure 6D, TC displays the shortest semicircle in the electrochemical impedance spectroscopy (EIS) plot due to its exceptional conductivity. The conductivity of pCN/TC/SBM is intermediate between that of pCN/SBM and TC. The CO yield significantly increases to 36.33 μmol⋅g^−1^⋅h^−1^, which is 4.36 times greater than that of pCN and 3.5 times higher than SBM, when the heterojunction is formed. The pCN/TC/SBM was very selective to produce CO and reached to 95.49 percent. *In situ* infrared spectroscopy can be employed to investigate the root cause.

Figure 6E illustrates the reaction mechanism, providing clarity on the reduction process. Photogenerated electrons over pCN under light irradiation migrate to TC until equilibrium is reached. As a result of the space charge generated on the pCN side, the band bends downward to form a Schottky junction. The photogenerated electrons from pCN are captured and prevented from recombining due to TC’s high conductivity, enhancing space charge separation and accelerating electron transport. The integration of the heterojunction with the Schottky junction enables efficient separation of space charges and much faster electron transit.

Table 1 summarises various types of Ti_3_C_2_ MXene/MOF composites used for various CO_2_ reduction and H_2_ production reactions. More research has been conducted for hydrogen production, however, only limited data was available for CO_2_ reduction applications. It can be seen that CO_2_ reduction to CO was significantly enhanced with Ti_3_C_2_ MXene coupling with MOF-derived materials. Similarly, for water splitting, the production of hydrogen was significantly enhanced in Ti_3_C_2_ MXenes-based MOF composites.

**TABLE 1:** An overview of Ti_3_C_2_ MXenes-based MOF photocatalyst for producing H_2_ and reducing CO_2_ is given.

Catalysts	Results	Ref.
MOF-based Co-Co LDH/Ti_3_C_2_T_x_	• CO = 1.25×10^4^ µmol h^−1^ g^−1^ • CH_4_ = 6.248 µmol h^−1^ • AQE = 0.92 %	(Chen et al., 2020)
Ti_3_C_2_/TiO_2_/UiO-66-NH_2_	• H_2_ = 1980 µmol h^−1^ g^−1^ • Formation of Schottky junction and heterojunctions	(Tian et al., 2019a)
Ti_3_C_2_/UiO-66-NH_2_	• H_2_ = 204 µmol h^−1^ g^−1^ • Formation of Schottky junction	(Tian et al., 2019b)
TiO_2/_Ti_3_C_2_-CoS_x_	• H_2_ = 0.95 mmol h^−1^ g^−1^ • CoS_x_ derived from ZIF-67	(Zhao et al., 2019)
Ti_3_C_2_/MIL-NH_2_	• H_2_ = 4383.1 µmol h^−1^ g^−1^	(Li et al., 2021)

## 4 Applications of Ti_3_C_2_-based MOF composites for hydrogen production

### 4.1 Mechanism of photocatalytic H_2_ production

**FIGURE 7:**
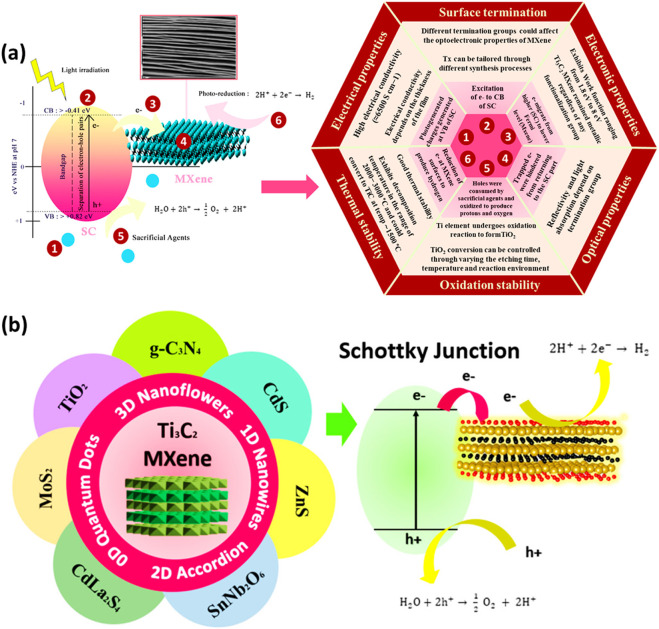
**(A)** Mechanism of H_2_ production over MXenes/semiconductor composites, **(B)** Ti_3_C_2_ MXenes coupled with different semiconductors and the process of charge separation. Reproduced with permission from (Sherryna and Tahir, 2021). Copyright 2021 Elsevier.

A low-carbon economy and the accomplishment of sustainable development objectives depend heavily on hydrogen as a clean and sustainable energy source. While there are other sustainable methods for producing hydrogen, two of the more common ones are electrocatalytic and photocatalytic water splitting (Zhang et al., 2024). Despite being environmentally benign and emitting no CO_2_, photocatalytic hydrogen production has a lower photocatalytic efficiency because light and catalysts are required. In photocatalytic hydrogen production, the hydrogen yield depends on three factors: the band position of the semiconductor, the band gap energy, and the efficiency of charge separation. Ti_3_C_2_ MXenes-based photocatalysts have been widely utilized in photocatalytic H_2_ production due to their remarkable properties. Figure 7A presents a schematic of hydrogen production over Ti_3_C_2_ MXenes with a semiconductor.

In photocatalysis, four steps are involved to complete the process which includes charge production under the light energy, charge separation within the bulk, oxidation and reduction reactions over the catalyst surface. In the first step, when the light energy which have enough power strikes the catalyst surface, it enables to generates electrons and holes. The number of charges depends on the band gap of the photocatalysts, light intensity and wavelength. During the excitations, charges (e^−^/h^+^) are produced at the valance band (VB) of the semiconductor and then electrons are moved to the conduction band (CB), which enables their separation. If the charges are successful to separate from the bulk surface to outer surface without recombination, then oxidation and reduction reactions occurs. In general, oxidation occur at VB position with the use of holes to produce protons and oxygen. However, production of hydrogen occurs at the CB position through the reduction of protons with electrons (Alfaifi et al., 2018; Shkrob and Sauer, 2004; Maeda, 2011; Jalil et al., 2021; Linsebigler et al., 1995).

The process of producing H_2_ through photocatalytic water-splitting involves two crucial stages. Equation 6 shows the oxidation process, whereas in the first stage oxidize water molecules to produce two protons and oxygen. Equation 7 describes the reduction process, in which protons and electrons are used to produce hydrogen.

$$H_{2}O+2h^{+}\;\to \;0.5O_{2}+2H^{+}\;\left(+0.81\text{ eV}\right)$$ (6)

$$2H^{+}+2e^{-}\;\to \;H_{2}\;\left(-0.42\text{ eV}\right)$$ (7)

The Ti_3_C_2_ MXenes have several attractive characteristics such as thermal stability, electrical conductivity, surface terminal groups, optical characteristics and others, which are beneficial to be coupled with any semiconductor to maximize photocatalytic efficiency. The charge transfer mechanism in the MXenes-based composite is accomplished by the semiconductors and metallic MXenes’ different work functions. Figure 7B illustrates the basic mechanism of water splitting through photocatalysis under light irradiation over various type of semiconductors. Typically, a Schottky junction forms when Ti_3_C_2_ is used as a cocatalyst with a semiconductor, aiding the separation of electron-hole pairs. When MXenes and the semiconductor are in close contact, a Schottky barrier is created, leading to strong interfacial charges at the metal-semiconductor interfaces. Upon light exposure, the semiconductor generates photogenerated charges. Excited electrons move to the semiconductor’s conduction band (CB), leaving photogenerated holes in the valence band (VB). MXenes, acting as electron acceptors, generally possess a larger work function and a lower Fermi level than the semiconductor. Electrons will transfer from the semiconductor with the higher Fermi level to the MXene until the Fermi levels align (Sherryna and Tahir, 2021).

### 4.2 MOF for photocatalytic H_2_ production

To enhance photocatalytic efficiency, MXenes work as cocatalysts with other semiconductors to create binary composites and heterojunctions. The electrical mobility of Ti_3_C_2_ MXene has sparked considerable attention in photocatalysis, primarily owing to its ability to facilitate adequate carrier segregation. In this development, Ti_3_C_2_ MXenes are frequently employed as catalysts in photocatalytic water splitting, which yields H_2_ higher hydrogen compared to pure semiconductor materials (Jacobs et al., 2011; Liu et al., 2019). Numerous research findings indicate that Ti_3_C_2_ is a highly effective co-catalyst in photocatalysis, enhancing the activity of semiconductors (Jacobs et al., 2011; Ibragimova et al., 2021). Because of the functional groups–O, and–F, –OH over the surface of Ti_3_C_2_, electrostatic adsorption is frequently used to modify semiconductors. The organic ligand of the MOF can more easily coordinate with metals because of its structure Hence, there is a high probability that the organic ligand in MOFs will form a bond with titanium in Ti_3_C_2_, resulting in a distinct interaction mechanism. This could have an impact on MOFs’ photocatalytic efficiency when combined with Ti_3_C_2_ (Liu et al., 2019; Ibragimova et al., 2021; Im et al., 2021).

Previously, synthesis and characterization of NH_2_-MIL-88 and MXenes/MOF composites were conducted and observed increased photocatalytic properties (Long et al., 2021). Ti_3_C_2_ MXene was synthesized using HF as the etching agent, while NH_2_ -MIL-88 MOF was produced via a hydrothermal method. Compared to pure MOF, the binary composite of the two materials exhibited higher light absorbance. NH_2_-MIL-88B shows light absorbance over the full spectrum ranging from 200 to 700 nm, effectively capturing both visible and UV light. The significant absorption in the UV range is due to the π-π* transition in the organic linker. When MXene is added to the composite, the light absorption rate increases above 667 nm. When more MXene is added, light absorption first rises but eventually falls. This is probably because too much MXene causes NH_2_-MIL-88B to take on an unpredictable shape and size. When compared to the original material, 0.25-MXene/NH_2_-MIL-88B band gap was reduced which exhibits notable light absorbance ability. In addition to this, charge separation efficiency was also increased using composites. The creation of a heterojunction can enhance the photocatalyst’s activity by promoting the separation of photo-generated electron-hole pairs. The PL spectrum intensity of the composite material 0.25-MXene/NH_2_-MIL-88B lies between those of its constituent parts, indicating that the inclusion of MXenes may promote NH_2_-MIL-88B electrons and holes separation to maximize the photocatalytic efficiency.

**FIGURE 8:**
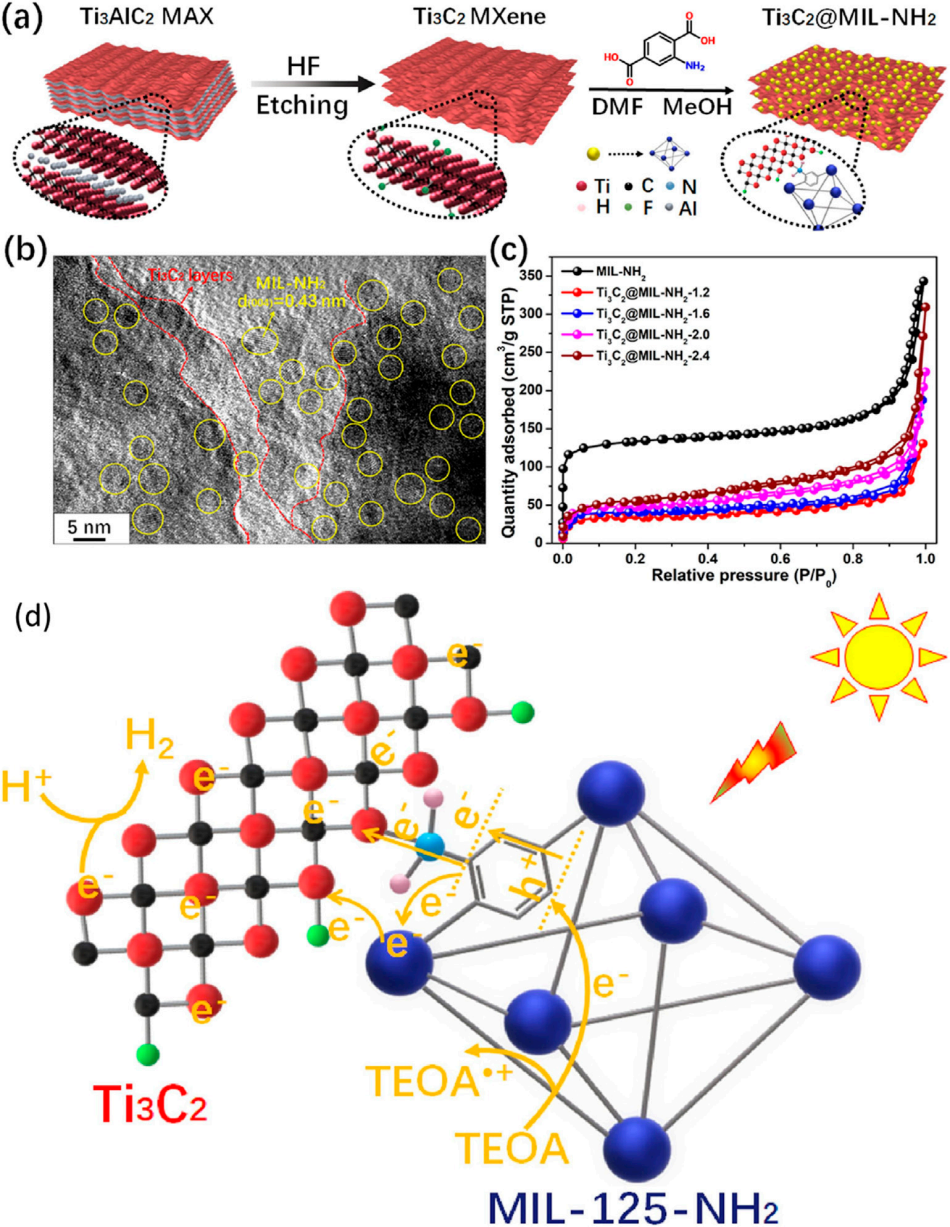
**(A)** Schematic illustration of Ti_3_C_2_/NH_2_-MIL-125 composite formation, **(B)** TEM images of the composites, **(C)** N_2_ adsorption-desorption analysis of Ti_3_C_2_/NH_2_-MIL-125 composite, **(D)** proposed mechanism of efficiency enhancement over Ti_3_C_2_/NH_2_-MIL-125 composite. Reproduced with permission from (Li et al., 2021). Copyright 2021 Elsevier.

Using Ti_3_C_2_-based MOF binary composites for photocatalytic hydrogen production is recently attained significant considerations. In recent work, Li and co-workers produced a Ti_3_C_2_-loaded MIL-NH_2_ composite and tested it for photocatalytic hydrogen production (Li et al., 2021). A schematic representation of the synthesis of Ti_3_C_2_ MXenes via HF etching of Ti_3_AlC_2_ and the subsequent production of the Ti_3_C_2_/MIL-NH_2_ composite is shown in Figure 8A. The Ti_3_C_2_ MXenes were produced using an HF etching agent, while the reaction was conducted for 72 h to get a 2D layered structure of Ti_3_C_2_ MXenes. The composite of Ti_3_C_2_/NH_2_-MIL was synthesized using the *in-situ* approach, by heating at 120°C for 84 h Ti_3_C_2_ exhibits an accordion-like layered MXenes configuration. Figure 8B shows the results of the composites in which both materials have good interface contact. The absorbance edge of the MIL-NH_2_ was 600 nm, which was increased to 800 nm when Ti_3_C_2_ was coupled with MIL-NH_2_. Furthermore, a band gap of 2.6 eV and a CB position of −0.76 eV were reported, which are beneficial to maximize the visible light absorbance and efficient hydrogen production. In addition to this, Ti_3_C_2_/NH_2_-MIL shows higher current density than using pure NH_2_-MIL under dark and solar light irradiation. These findings were further confirmed by EIS, in which a lower recombination process of photo-generated carriers was obtained with Ti_3_C_2_-MXene-loaded NH_2_-MIL.

The N_2_ adsorption-desorption isotherms were further used to understand the surface properties and the results are shown in Figure 8C. The BET surface area was decreased with Ti_3_C_2_-loaded NH_2_-MIL samples, which confirmed that it is not important in photocatalytic applications. Enhancing carrier segregation and transfer efficiency is well recognized to always be advantageous for photocatalytic process activation (Li et al., 2021). In simulated sunlight, the hybrid Ti_3_C_2_/MIL-NH_2_ significantly enhances H_2_ generation. On the other hand, the Ti_3_C_2_-loaded MIL-NH_2_ hybrids show significantly increased activity which was due to prevented charge carrier recombination and higher visible light absorbance. The highest hydrogen yield of 4383.1 μmol h^−1^g^−1^ was reported with the optimized Ti_3_C_2_/MIL-NH_2_-1.6 composite. This productivity was about five times higher than a physical combination of MIL-NH_2_ and Ti_3_C_2_ samples, and it was six times higher than that of MIL-NH_2_. More intriguing results included the discovery of an apparent quantum efficiency of 3.140 percent for Ti_3_C_2_-MIL-NH_2_-1.6 composite. Nonetheless, after consecutive four cycles, the H_2_ generation rate over Ti_3_C_2_-MIL-NH_2_-1.6 did not substantially drop, indicating good photostability of the Ti_3_C_2_-supported MOF composite. Figure 8D shows the efficiency enhancement approach over the Ti_3_C_2_/MIL-NH_2_ under visible light irradiation. The charges were produced over the NH_2_-MIL under visible light and were trapped by the Ti_3_C_2_ MXenes, which resulted in efficient charge carrier separation and improved hydrogen production. By using terephthalic acid and replacing MIL-125-NH_2_ with MIL-125, Ti and N’s cooperation can operate. When compared to Ti_3_C_2_/MIL-NH_2_, the resulting Ti_3_C_2_/MIL shows significantly reduced activity, according to the photocatalytic H_2_ generation rate.

These results can be further explained based on the various MXenes characteristics. Ti_3_C_2_ MXenes due to 2D structure can improve the interaction between water molecules and the photocatalyst and have a high hydrophilicity (Sharma et al., 2022c). For example, Tian et al. altered Ti_3_C_2_ nanosheets for an effective photocatalytic hydrogen evolution reaction by adding a porous, water-stable Zr-based UiO-66-NH_2_ (HER). Compared to pure UiO-66-NH_2_ and the Ti_3_C_2_, the composite of UiO-66-NH_2_/TiC_3_C_2_ displayed a lower PL intensity, suggesting a decreased recombination rate. The results showed that just 25.6 μmol h^−1^ g^−1^ of H_2_ was evolved using pristine UiO-66-NH_2_ MOF. Furthermore, Ti_3_C_2_ nanosheets produced 204 µmol H_2_ h^−1^ g^−1^ of hydrogen, eight times the photocatalytic activity of the composite (TU10). The formation of a Schottky connection between Ti_3_C_2_ and UiO-66-NH_2_ was responsible for efficient charge carrier separation and increased lifetime of the electrons. Due to its extremely positive Fermi level and low Gibbs free energy, the O-terminated Ti_3_C_2_ can capture electrons from the MOF for the generation of H_2_. This is the major reason for the efficient migration and separation of charge carriers (Tian et al., 2019b).

In summary, binary Ti_3_C_2_-supported MOF composites are beneficial to maximize the charge carrier separation and higher visible light absorbance, which are beneficial to stimulate photocatalytic hydrogen production.

### 4.3 MOF-based composites for photocatalytic H_2_ production

The performance of the binary composite can be further enhanced by adding third materials to construct a ternary composite, which can exhibit excellent photocatalytic performance due to their elevated Fermi levels, outstanding conductivity, and efficient carrier transport properties (Tian et al., 2019b; You et al., 2021). Heterostructures can be formed using MXenes and semiconductor combinations, establishing a Schottky junction at the material interface. The addition of third materials with the binary junction has the potential to enhance the migration of photo-generated electrons (Hong L.-f. et al., 2020). Enhancing the effective facilitation of charge-carrier transport often requires the strategic optimisation of interfacial architecture. This involves meticulous component selection and minimising imperfections at the interface. The combination of Ti_3_C_2_ MXenes, known for their outstanding conductivity, with porous MOFs featuring fully utilized photoactive sites is anticipated to accelerate the migration of photo-generated electrons. This augmentation is poised to enhance the efficiency of extended photocatalytic hydrogen generation (Tian et al., 2019b; You et al., 2021; Hong L.-f. et al., 2020).

**FIGURE 9:**
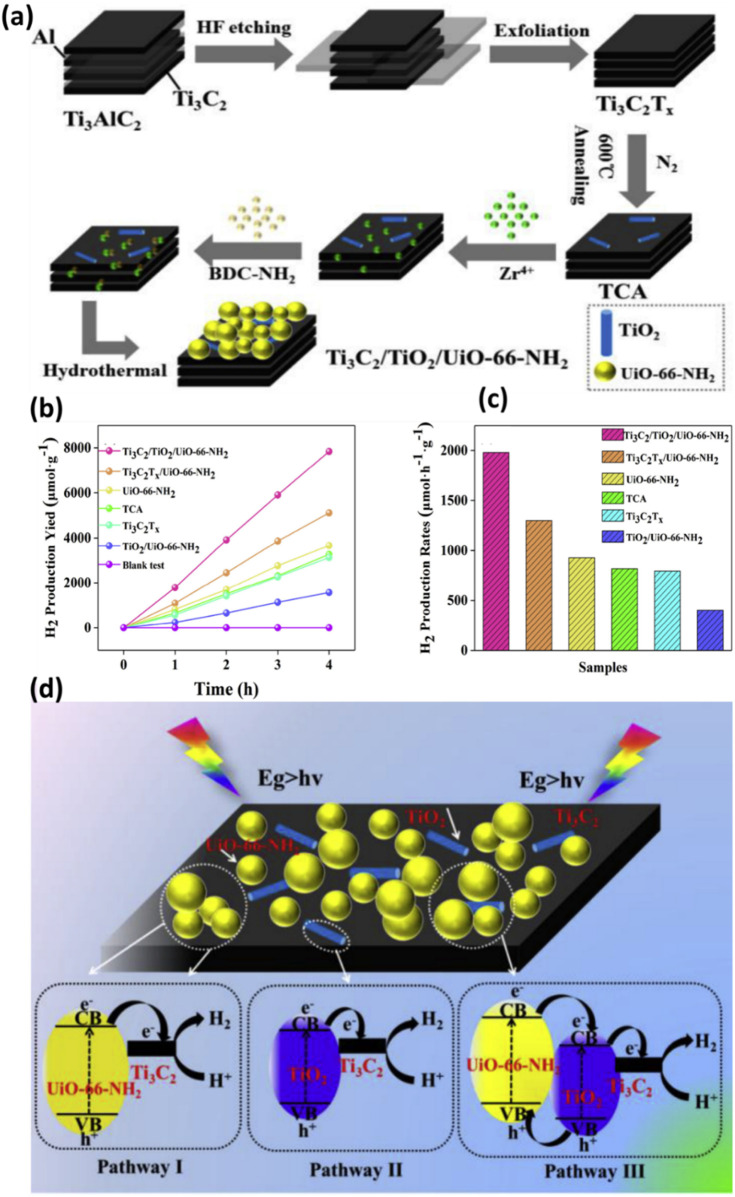
**(A)** A suggested chemical process is schematically depicted for the synthesis of Ti_3_C_2_-TiO_2_-UiO-66-NH_2_, **(B,C)** Performance analysis of pure and Ti_3_C_2_-TiO_2_-UiO-66-NH_2_ composite materials, **(D)** Proposed mechanism of photocatalytic charge separation and efficiency enhancement. Reproduced with permission from (Tian et al., 2018). Copyright 2018 Elsevier.

There are only limited reports available on the use of ternary composite to maximize the photocatalytic hydrogen production efficiency. In recent work, Tian et al. (Tian et al., 2018) thoroughly analysed Ti_3_C_2_/TiO_2_/UiO-66-NH_2_ for its potential in photocatalytic hydrogen generation. As a typical Metal-Organic Framework (MOF), the photo-responsive photocatalyst UiO-66-NH_2_ was studied with Ti_3_C_2_T_x_ to promote interfacial charge transfer. In this process, TiO_2_ layers, or TCA, were initially created by annealing the Ti_3_C_2_T_x_ MXenes. The one-step hydrothermal method employed to electrostatically adsorb UiO-66-NH_2_ onto the annealed Ti_3_C_2_T_x_ surfaces is illustrated in Figure 9A. Compared to Ti_3_C_2_T_x_ and UiO-66-NH_2_, the Ti_3_C_2_-based TiO_2_/UiO-66-NH_2_ composite exhibited superior photocatalytic activity for H_2_ production. TCA served as a platform and stored charge carriers, prolonging the lifetime of co-catalysts and UiO-66-NH_2_. Interestingly, after HF etching, Ti_3_C_2_T_x_ exhibited accordion-like structures, indicating the successful removal of the Al layers in Ti_3_AlC_2_. Despite its low concentration, TCA displayed a layered structure similar to pure Ti_3_C_2_T_x_ without noticeable TiO_2_. Ti_3_C_2_T_x_ was tightly wrapped in UiO-66-NH_2_, resulting in significant agglomeration. In contrast, Ti_3_C_2_-assisted TiO_2_ and UiO-66-NH_2_ composite showed higher charge separation with enhanced accessibility to reactive sites.

Photocatalytic H_2_ generation was conducted under artificial sunlight and the results are shown in Figures 9B,C. Ti_3_C_2_/TiO_2_/UiO-66-NH_2_ outperforms other combinations with the maximum hydrogen yield (7,840 μmol g^−1^). This is because of its synergistic effect and increased light-collecting ability. A H_2_ yield of 1980 μmol h^−1^ g^−1^ was obtained by adding TCA to the H_2_ evolution rates. This is 1.5 times higher than Ti_3_C_2_T_x_-UiO-66-NH_2_ and 2.1 times higher than pure UiO-66-NH_2_, respectively. The stability test, which lasted 12 h and revealed a modest decrease in HER activity after three cycles, proved the Ti_3_C_2_-TiO_2_-UiO-66-NH_2_ structure’s longevity. Like this, TiO_2_ layers were originally produced by annealing Ti_3_C_2_ MXenes in the N_2_ environment. Ti_3_C_2_-TiO_2_-UiO-66-NH_2_ was the composite that was produced after UiO-66-NH_2_ was applied to the surface of Ti_3_C_2_T_x_ layers. Compared to its Ti_3_C_2_-UiO-66-NH_2_ cousin, the photocatalytic output was boosted 1.5 times by annealing Ti_3_C_2_ to create TiO_2_. This is because the Mott-Schottky plots showed a negative shift due to the production of TiO_2_, which clarified stronger reducibility and an increased electron/hole pair separation. The first and second paths, as shown in Figure 9D, traverse the Schottky junction between Ti_3_C_2_ nanosheets containing TiO_2_ and UiO-66-NH_2_, respectively. Type II heterojunction formation among TiO_2_ and UiO-66-NH_2_ is the third process involves, in which electron transfer from the CB of TiO_2_ to Ti_3_C_2_ MXenes (Tian et al., 2019a). Interestingly, Li et al. (Li et al., 2021) managed to successfully synthesized Ti/TiC MXenes with MIL-NH_2_ through *in-situ* growth, resulting in the formation of a Ti_3_C_2_/MIL-NH_2_ composite. When comparing the *in-situ* grown Ti_3_C_2_/MIL-NH_2_ to the usual physical mixing with MIL-NH_2_, the H_2_ generation rate was five times higher. This is a result of the Ti_3_C_2_ and MIL-NH_2_ coming into close contact. Furthermore, Ti_3_C_2_/MIL-NH_2_ showed exceptional stability; even after four cycles, there was hardly any decrease in the rate of H_2_ generation. The promising results were due to the presence of Ti/MXene with MOF. Consequently, the Ti_3_C_2_ becomes more electron-rich, efficiently adsorbing H^+^ and reducing it to H_2_. However, despite the paucity of research, 2D Ti_3_C_2_ sheets combined with MOF composites exhibit significant potential as a photocatalyst for effective chemicals and other products and need further investigation in the coming years. Table 2 shows the summary of TI3C2/MOF composites for photocatalytic hydrogen production.

**TABLE 2:** Comparison of photocatalytic hydrogen evolution rate by the use of different TiC MXene-based MOF Composites.

Photocatalyst	Sacrificial agent	Illumination source	HER	Ref.
Ti_3_C_2_/MIL-NH_2_	TEOA	300 W Xe lamp	4383.1 μmol h^−1^ g^−1^	Li et al. (2021)
Ti_3_C_2_T_x_/UiO-66-NH_2_	Na_2_S and Na_2_SO_3_	300 W Xe lamp	1,320 μmol h^−1^ g^−1^	Tian et al. (2018)
Ti_3_C_2_/TiO_2_/UiO-66-NH_2_	Na_2_S and Na_2_SO_3_	300 W Xe lamp	1980 μmol h^−1^ g^−1^	Tian et al. (2018)
TiO_2_-Ti_3_C_2_-CoSx	Methanol	300 W Xe lamp	0.95 mmol h^−1^ g^−1^	Zhao et al. (2019)

### 4.4 MOF-derived materials for photocatalytic H_2_ production

Instead of using MOF, their derived materials can be coupled with other semiconductors and materials to enhance photocatalytic efficiency and stability under extreme reaction temperatures. The MOF-derived materials provide higher specific surface area and nanostructure properties, which make them superior compared to conventional methods of materials synthesis (Fan et al., 2024). For example, ZIF-67-templated CoS_x_ coupled with TiO_2_/Ti_3_C_2_ was investigated by Zhao et al. (Zhao et al., 2019) for photocatalytic H_2_ production. The ZIF-67-templated CoSx’s porous morphology improved charge transfer segregation and utilization efficiency in addition to the shape of TiO_2_ nanoparticles. In addition to increasing heterostructure conductivity, the conductive Ti_3_C_2_ MXenes may also improve photo-generated carrier transfer. Consequently, the photocatalytic activity of the resulting TiO_2_-Ti_3_C_2_-CoSx heterostructure exhibited notable enhancements. The hydrogen production activity of pure TiO_2_ is measured at 0.14 mmol/h/g. However, incorporating just 1% of ZIF-67-templated CoSx led to a significant enhancement in photocatalytic activity. As the concentration of CoSx increased, so did the rates of hydrogen production by TiO_2_-CoS_x_. The optimal H_2_ generation occurred at 0.54 mmol/h/g, representing a 2.8-fold increase compared to pure TiO_2_, achieved after the molar ratio of CoSx reached 1%. Although the rate of hydrogen yield was decreased with higher CoSx loading, yet higher H_2_ was obtained than pure TiO_2_.

Pure TiO_2_ produces H_2_ of 0.14 mmol/h/g. On the other hand, a small addition of 1 per cent ZIF-67-templated CoSx increased photocatalytic activity significantly. The rates at which TCx generated hydrogen increased with the content of CoSx. At a molar ratio of 1 percent for CoSx, the best H_2_ generation was seen at 0.54 mmol/h/g, or 2.8 times that of pure TiO_2_. While the activity of hydrogen creation remained higher than that of pure TiO_2_, the rate of hydrogen synthesis reduced as the concentration of CoSx rose. In a similar vein, the interaction of TiO_2_-Ti_3_C_2_ with Ti_3_C_2_ demonstrated that the maximum photocatalytic activity was obtained at a concentration of 0.5% Ti_3_C_2_, likely due to the high conductivity of Ti_3_C_2_. As the quantity of black Ti_3_C_2_ was raised, the colour of the sample darkened, resulting in a drop in optical absorption in TiO_2_ and a reduction in photocatalytic efficiency. A detailed analysis contrasting pure TiO_2_ with the will provide a more complete picture of how ZIF-67-derived CoSx and Ti_3_C_2_ affect TiO_2_ photocatalytic activity. In the presence of conductive Ti_3_C_2_ and porous CoSx produced from ZIF-67, the photocatalytic HER rate increased from 0.14 to 0.54 and 0.33 mmol/h/g, respectively. Remarkably, this value was increased to 0.95 mmol/h/g by the synergistic effect of co-loading CoSx and Ti_3_C_2_. For the photocatalytic hydrogen evolution process, all three materials exhibited remarkable durability. There was no noticeable decrease in the rate of H_2_ evolution for any of the three materials during five cycles in 15 h under UV irradiation. Significant improvements were made to the cocatalyst for the hydrogen evolution reaction and their photocatalytic H_2_-production activities by adding highly conductive Ti_3_C_2_ and a highly porous MOF-templated CoSx. This work provides guidelines for designing and manufacturing efficient photocatalysts with good charge carrier transport and utilization efficiency for a range of energy and environmental applications.

## 5 Comparative Assessment and challenges

### 5.1 Overview and challenges

**FIGURE 10:**
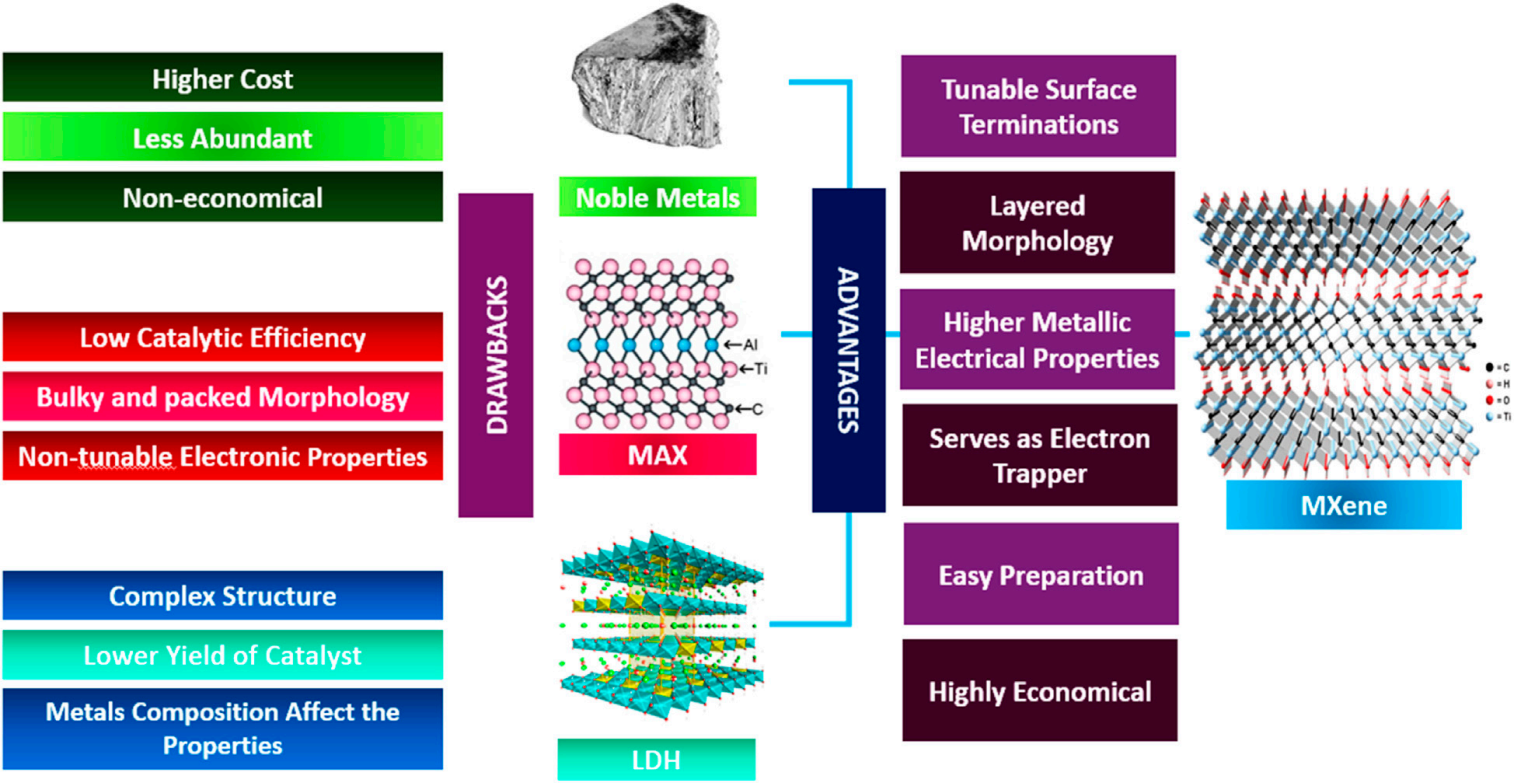
Comparative analysis of MXenes benefits and limitations of other co-catalysts (Fan et al., 2022).

This section highlights the challenges and comparisons of Ti_3_C_2_ MXenes with alternative materials, as illustrated in Figure 10. Ti_3_C_2_T_x_ MXenes offer enhanced electron trapping and a higher work function, which provide advantages for maximizing solar fuel generation. However, the control of termination functional groups in Ti_3_C_2_T_x_ MXenes presents a challenge for its use as a photoactivity enhancer. The electrical and optical properties are significantly influenced by tunable termination groups (Tx), with studies indicating that the terminal groups in MXenes materials can determine their metallic or semiconducting properties (Khazaei et al., 2017). Variations in the work function value can lead to differences in termination properties, which can affect Ti_3_C_2_Tx MXenes photocatalytic efficiency (Chertopalov and Mochalin, 2018). Theoretical investigations revealed a work function range of 5.75–6.25 eV for -O terminated Ti_3_C_2_T_x_ MXenes, while -OH terminated Ti_3_C_2_T_x_ MXenes were found to have a work function ranging from 1.6 to 2.8 eV (Sherryna and Tahir, 2022b). Photocatalysis studies suggest that maximizing the conversion of solar energy to hydrogen is best achieved by ensuring a substantial variation in the metalwork function between the main catalyst and the metal co-catalyst (Fajrina and Tahir, 2019). Ti_3_C_2_T_x_ MXenes stand out among metallic materials, including noble metals, due to their unique capability to adapt their work function to specific application requirements. The disparity between theoretical analysis and experimental evaluation arises from the difficulty in achieving a perfect single termination group. Additionally, controlling the distribution of termination groups in MXene materials poses a significant challenge (Naguib et al., 2011). Hence, synthesizing mixed terminating Ti_3_C_2_T_x_ MXene can yield diverse outcomes and catalytic efficiencies. Nevertheless, most research affirms that employing Ti_3_C_2_T_x_ MXene as a co-catalyst undeniably enhances the semiconductor’s capabilities, yielding positive outcomes (Tahir, 2021a; Tahir, 2021b; Khan and Tahir, 2021; Tahir and Tahir, 2020). The superior electrical conductivity and distinctive qualities make these materials stand out as potential replacements for costly and inefficient co-catalysts.

### 5.2 Addition of metals

Transition metals are important for improving the photocatalytic efficiency because of their distinct electronic structures and capacity to create variety of oxidation states. The higher photocatalytic efficiency of NH_2_-MIL-125-Ti MOF for hydrogen production was achieved by incorporating various transition metals such as Co, Cu and Ni. The multi-metal sites speed up the separation of charge carriers and greatly improve optical absorption by d-d transitions when M^2+^ ions are coordinated with MOF. This increases the activity of solar light-driven H_2_ generation (Karthik et al., 2020). Noble metals like Ag, Au, and Pt share characteristics with Ti_3_C_2_T_x_ MXene and are considered excellent metal co-catalysts in energy conversion due to their ability to form the Schottky barrier and exhibit surface plasmon resonance (SPR) effects. However, their high costs remain a significant drawback for their practical use in photocatalytic applications (Afroz et al., 2018). Ti_3_C_2_T_x_ MXene emerges as a cost-effective alternative to noble metals, offering comparable functionality at a lower material cost, making it commercially feasible for large-scale semiconductor fabrication. Its work function closely aligns with noble metals (3.9 to 6.25 eV), and both share functional qualities that enhance carrier dynamics and facilitate electron transfer, forming a potential energy barrier in metal-semiconductor interfaces.

Ti_3_C_2_T_x_ MXene features a structurally layered, two-dimensional design often likened to an accordion-like form. This unique structure offers a significantly larger surface area compared to the bulky, compact, and densely layered Ti_3_AlC_2_ MAX precursor (Tahir et al., 2021b). Unlike the densely packed layers of MAX material, Ti_3_C_2_Tx MXene loosely arranged layers with larger intervals provide effective sites for attaching other semiconductors. This loose structure enhances interfacial contact, speeding up redox reactions and facilitating quick charge transfer. Ti_3_C_2_T_x_ MXene also prevents agglomeration, offering a stable foundation for the uniform dispersion of particle semiconductors, and its well-defined layers with space intervals support redox reactions in both inner and outer layers (Tahir, 2021b). While several studies indicate that Ti_3_AlC_2_ MAX may help convert solar energy into fuel, Ti_3_C_2_T_x_ MXene’s catalytic efficiency still outperforms Ti_3_AlC_2_ MAX (Tasleem et al., 2020). As mentioned earlier, the surfaces of Ti with functional groups such as -OH, -F, and -O can be terminated through chemical etching, eliminating the Al layer. These surface terminations, known for increased hydrophilicity, exhibit superior interaction with water molecules. Notably, functional groups like -OH facilitate hydrogen generation through reduction from water capture, a capability not shared by Ti_3_AlC_2_ MAX. Layered Double Hydroxide (LDH), a frequently utilized co-catalyst in various material research, exhibits a flexible and diverse compositional matrix. Like MXene materials, LDH has several layers that can be easily adjusted to change its electrical properties by changing the kinds of metal cations and anions that are present in its matrix structures (Sherryna et al., 2021).

### 5.3 Addition of carbon materials

To increase photocatalytic efficiency, meal-free carbon compounds can be employed as cocatalysts with the metal-organic framework (MOF). As discussed previously, although, MOF have several promising characteristics such as a porous structure with high surface area and tuneable pore structure. However, in photocatalysis, the main challenge is the charge recombination within the semiconductor during the photocatalysis process. In addition to Ti_3_C_2_ MXenes, carbon materials such as graphene can be used as a cocatalyst to prevent charge recombination rate. Coupling MOFs with graphene can construct a good interface interaction and it can reduce charge recombination and makes charge carrier separation and transportation easier. For example, Karthik et al. (Karthik et al., 2018) investigated the role of graphene with MOF for photocatalytic hydrogen production. The strong π−π interaction between MOF and rGO was beneficial to construct good interface interaction and it was responsible for the efficient transfer and separation of photoinduced electron−hole pairs, resulting in a steady and increased production of hydrogen. In another development, charge transfer was regulated in bimetallic ZnCd-ZIF-8 with the use of graphene oxide. The π−π interactions between GO and ZnCd-ZIF-8 were responsible for the efficient separation of electron−hole charges, which resulted in significantly higher hydrogen production. It was also recommended that the stability of MOF be enhanced by using bimetallic MOF such as ZnCd-MOF (Gonuguntla et al., 2023). In contrast, Ti_3_C_2_T_x_ MXene emerges as the ideal option due to its easy fabrication, unique structural features, economic feasibility, and outstanding photocatalytic efficiency.

## 6 Conclusion and outlook

In summary, there is ongoing research on the use of metallic-organic framework (MOF) due to their unique advantages such as porous structure and higher specific surface area. MOFs have structural flexibility due to the use of a large variety of precursor building blocks. The lower electrical conductivity and photocatalytic efficiency of MOFs can be enhanced by using higher conductive materials as the cocatalysts. Since the discovery of Ti_3_C_2_ in 2011, the family of 2D transition metal carbides carbonitrides, and nitrides—collectively known as MXenes—has garnered significant attention in the academic community. MXenes are particularly promising due to their excellent electrical conductivity, broad and controllable layer spacing, tunable surface functional groups, and high real density. This review paper explores the effectiveness of documented TiC MXene-based MOF nanohybrids and nanocomposites for photocatalytic CO_2_ reduction and water-splitting applications.

The expanded surface area of TiC MXenes makes it widely used as a co-catalyst in photocatalysis. The compelling characteristics of TiC MXenes have led to their exploration in combination with Metal-Organic Frameworks (MOFs). Reported successes in these studies are attributed to the synergistic enhancements achieved through their combination. As a result, common challenges associated with inefficient photocatalysts, such as rapid recombination rates of electron-hole pairs and suboptimal charge-carrier separation, have been overcome, leading to heightened efficiency under illumination.

Scalability issues with TiC with MOFs, however, make it necessary to make efficient use of TiC MXenes’ co-catalytic properties in order to produce high-efficiency photocatalysts. The characteristics and paths of charge transfer in TiC MXene-based MOF composites are largely determined by the surface termination groups of the MXenes. There is little overlap between theory and experiment when studying MXenes in photocatalysis. Understanding the mechanisms underlying interfacial charge transfer will be crucial to the advancement of TiC MXene-based composites to increase both photoactivity and stability.

Significant promise for increasing photocatalytic activity has been demonstrated by the development of TiC MXene-based MOF photocatalysts. This improvement is credited to synergistic effects, including enhanced stability and the establishment of a Schottky junction, creating a built-in electric field. While promising results have been achieved in solar fuel production with TiC-based MOF composites, comprehensive studies are necessary. Therefore, more research on TiC with other MOFs is advised, especially focusing on photocatalytic CO_2_ reduction and water-splitting reactions.

**FIGURE 11:**
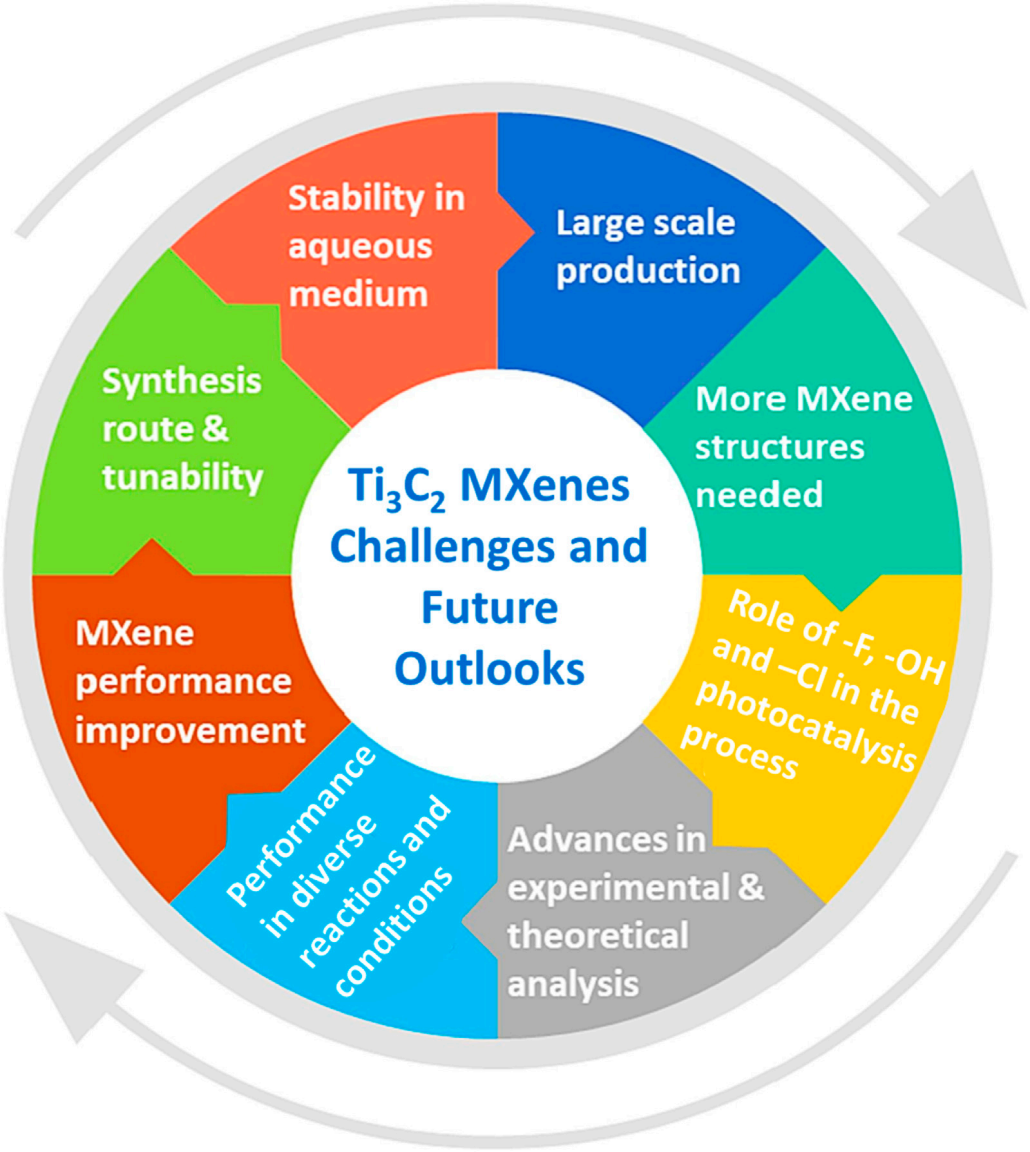
Recent progress, challenges and future recommendations.

The present challenges and suggestions for future research into Ti_3_C_2_ MXenes for various applications are summarized in Figure 11. TiC MXene-based MOF composites and hybrids need further thought and investigation. Even if the initial results seem promising, more investigation is needed to provide the scientific community with a deeper understanding of scalability and useful applications. Despite several benefits of MOFs, they have limitations of lower stability and lower charge transfer ability. The metals ions with higher valence state such as Ti^4+^, Zr^4+^, Ln^3+^, and Al^3+^ can be utilized to produce MOFs with their higher thermal stability. The structural tuning and with the selection of new synthesis methods, light absorbance and electrical properties can be improved. Although Ti_3_C_2_ MXenes have limited applications on their own, they are commonly used in conjunction with various semiconductors, including metal-organic frameworks (MOFs), for photocatalytic energy and environmental applications. Their superior conductivity and surface area lead to unprecedented improvements in efficiency and sustainability in hydrogen production and CO_2_ reduction. When used as cocatalysts, MXenes composites can enhance charge separation efficiency and improve solar energy utilization.

## References

[B1] Afroz K. Moniruddin M. Bakranov N. Kudaibergenov S. Nuraje N. (2018). A heterojunction strategy to improve the visible light sensitive water splitting performance of photocatalytic materials. J. Mater. Chem. A 6, 21696–21718. 10.1039/c8ta04165b

[B2] Alfaifi B. Y. Ullah H. Alfaifi S. Tahir A. A. Mallick T. K. (2018). Photoelectrochemical solar water splitting: from basic principles to advanced devices. Veruscript Funct. Nanomater. 2, BDJOC3. 10.22261/fnan.bdjoc3

[B3] Alhabeb M. Maleski K. Anasori B. Lelyukh P. Clark L. Sin S. (2017). Guidelines for synthesis and processing of two-dimensional titanium carbide (Ti_3_C_2_T_x_ MXene). Chem. Mater. 29, 7633–7644. 10.1021/acs.chemmater.7b02847

[B4] Alli Y. A. Bamisaye A. Nancy P. Zachariah S. M. Oladoye P. O. Bankole O. M. (2024). MXene composites: properties, synthesis and its emerging application in rechargeable batteries. J. Energy Storage 77, 109954. 10.1016/j.est.2023.109954

[B5] Anasori B. Lukatskaya M. R. Gogotsi Y. (2017). 2D metal carbides and nitrides (MXenes) for energy storage. Nat. Rev. Mater. 2, 16098. 10.1038/natrevmats.2016.98

[B6] Anasori B. Xie Y. Beidaghi M. Lu J. Hosler B. C. Hultman L. (2015). Two-dimensional, ordered, double transition metals carbides (MXenes). ACS Nano 9, 9507–9516. 10.1021/acsnano.5b03591 26208121

[B7] Balcı E. Akkuş Ü. Ö. Berber S. (2017). Band gap modification in doped MXene: Sc2CF2. J. Mater. Chem. C 5, 5956–5961. 10.1039/c7tc01765k

[B8] Balcı E. Akkuş Ü. Ö. Berber S. (2018). Controlling topological electronic structure of multifunctional MXene layer. Appl. Phys. Lett. 113, 083107. 10.1063/1.5042828

[B9] Bansal S. Chaudhary P. Sharma B. B. Saini S. Joshi A. (2024). Review of MXenes and their composites for energy storage applications. J. Energy Storage 87, 111420. 10.1016/j.est.2024.111420

[B10] Bao L. Jia Y. Ren X. Liu X. Dai C. Ali S. (2024). Cr dopants and S vacancies in ZnS to trigger efficient photocatalytic H_2_ evolution and CO_2_ reduction. J. Mater. Sci. and Technol. 199, 75–85. 10.1016/j.jmst.2024.01.094

[B11] Barton Cole E. Lakkaraju P. S. Rampulla D. M. Morris A. J. Abelev E. Bocarsly A. B. (2010). Using a one-electron shuttle for the multielectron reduction of CO_2_ to methanol: kinetic, mechanistic, and structural insights. J. Am. Chem. Soc. 132, 11539–11551. 10.1021/ja1023496 20666494

[B12] Berdiyorov G. R. (2015). Effect of surface functionalization on the electronic transport properties of Ti_3_C_2_ MXene. EPL Europhys. Lett. 111, 67002. 10.1209/0295-5075/111/67002

[B13] Bosch M. Zhang M. Zhou H.-C. (2014). Increasing the stability of metal-organic frameworks. Adv. Chem. 2014, 1–8. 10.1155/2014/182327

[B14] Burtch N. C. Jasuja H. Walton K. S. (2014). Water stability and adsorption in metal–organic frameworks. Chem. Rev. 114, 10575–10612. 10.1021/cr5002589 25264821

[B15] Cavka J. H. Jakobsen S. Olsbye U. Guillou N. Lamberti C. Bordiga S. (2008). A new zirconium inorganic building brick forming metal organic frameworks with exceptional stability. J. Am. Chem. Soc. 130, 13850–13851. 10.1021/ja8057953 18817383

[B16] Chaudhuri K. Wang Z. Alhabeb M. Maleski K. Gogotsi Y. Shalaev V. (2019). Optical properties of MXenes, 2D metal carbides and nitrides (MXenes). Springer International Publishing, 327–346.

[B17] Chen W. Han B. Xie Y. Liang S. Deng H. Lin Z. (2020). Ultrathin Co-Co LDHs nanosheets assembled vertically on MXene: 3D nanoarrays for boosted visible-light-driven CO_2_ reduction. Chem. Eng. J. 391, 123519. 10.1016/j.cej.2019.123519

[B18] Chertopalov S. Mochalin V. N. (2018). Environment-sensitive photoresponse of spontaneously partially oxidized Ti_3_C_2_ MXene thin films. ACS Nano 12, 6109–6116. 10.1021/acsnano.8b02379 29883092

[B19] Colombo V. Galli S. Choi H. J. Han G. D. Maspero A. Palmisano G. (2011). High thermal and chemical stability in pyrazolate-bridged metal–organic frameworks with exposed metal sites. Chem. Sci. 2, 1311–1319. 10.1039/c1sc00136a

[B20] Corma A. García H. Llabrés i Xamena F. X. (2010). Engineering metal organic frameworks for heterogeneous catalysis. Chem. Rev. 110, 4606–4655. 10.1021/cr9003924 20359232

[B21] Costentin C. Robert M. Savéant J.-M. (2013). Catalysis of the electrochemical reduction of carbon dioxide. Chem. Soc. Rev. 42, 2423–2436. 10.1039/c2cs35360a 23232552

[B22] Cui Y. Yue Y. Qian G. Chen B. (2012). Luminescent functional metal–organic frameworks. Chem. Rev. 112, 1126–1162. 10.1021/cr200101d 21688849

[B23] De S. Acharya S. Sahoo S. Shim J.-J. Nayak G. C. (2022). From 0D to 3D MXenes: their diverse syntheses, morphologies and applications. Mater. Chem. Front. 6, 818–842. 10.1039/d2qm00002d

[B24] Deng H. Grunder S. Cordova Kyle E. Valente C. Furukawa H. Hmadeh M. (2012). Large-pore apertures in a series of metal-organic frameworks. Science 336, 1018–1023. 10.1126/science.1220131 22628651

[B25] Dillon A. D. Ghidiu M. J. Krick A. L. Griggs J. May S. J. Gogotsi Y. (2016). Highly conductive optical quality solution-processed films of 2D titanium carbide. Adv. Funct. Mater. 26, 4162–4168. 10.1002/adfm.201600357

[B26] Dong L. Kumar H. Anasori B. Gogotsi Y. Shenoy V. B. (2017). Rational design of two-dimensional metallic and semiconducting spintronic materials based on ordered double-transition-metal MXenes. J. Phys. Chem. Lett. 8, 422–428. 10.1021/acs.jpclett.6b02751 28036178

[B27] Eddaoudi M. Kim J. Rosi N. Vodak D. Wachter J. O'Keeffe M. (2002). Systematic design of pore size and functionality in isoreticular MOFs and their application in methane storage. Science 295, 469–472. 10.1126/science.1067208 11799235

[B28] Enyashin A. N. Ivanovskii A. L. (2013). Two-dimensional titanium carbonitrides and their hydroxylated derivatives: structural, electronic properties and stability of MXenes Ti3C2−xNx(OH)2 from DFTB calculations. J. Solid State Chem. 207, 42–48. 10.1016/j.jssc.2013.09.010

[B29] Fajrina N. Tahir M. (2019). A critical review in strategies to improve photocatalytic water splitting towards hydrogen production. Int. J. Hydrogen Energy 44, 540–577. 10.1016/j.ijhydene.2018.10.200

[B30] Fan W. K. Sherryna A. Tahir M. (2022). Advances in titanium carbide (Ti_3_C_2_T_x_) MXenes and their metal-organic framework (MOF)-Based nanotextures for solar energy applications: a review. ACS Omega 7, 38158–38192. 10.1021/acsomega.2c05030 36340125 PMC9631731

[B31] Fan W. K. Tahir M. (2022). Recent advances on cobalt metal organic frameworks (MOFs) for photocatalytic CO_2_ reduction to renewable energy and fuels: a review on current progress and future directions. Energy Convers. Manag. 253, 115180. 10.1016/j.enconman.2021.115180

[B32] Fan W. K. Tahir M. Alias H. Mohamed A. R. (2024). Well-Designed morphology regulated ZIF-67 derived 0D/1D Co_3_O_4_@TiO_2_ NWs integrated with *in-situ* grown Ni/Co-Active metals for Low-Temperature driven CO_2_ methanation. Fuel 357, 130024. 10.1016/j.fuel.2023.130024

[B33] Farha O. K. Eryazici I. Jeong N. C. Hauser B. G. Wilmer C. E. Sarjeant A. A. (2012). Metal–organic framework materials with ultrahigh surface areas: is the sky the limit? J. Am. Chem. Soc. 134, 15016–15021. 10.1021/ja3055639 22906112

[B34] Feng D. Gu Z.-Y. Li J.-R. Jiang H.-L. Wei Z. Zhou H.-C. (2012). Zirconium-metalloporphyrin PCN-222: mesoporous metal–organic frameworks with ultrahigh stability as biomimetic catalysts. Angew. Chem. Int. Ed. 51, 10307–10310. 10.1002/anie.201204475 22907870

[B35] Férey G. Mellot-Draznieks C. Serre C. Millange F. Dutour J. Surblé S. (2005). A chromium terephthalate-based solid with unusually large pore volumes and surface area. Science 309, 2040–2042. 10.1126/science.1116275 16179475

[B36] Fujita M. Kwon Y. J. Washizu S. Ogura K. (1994). Preparation, clathration ability, and catalysis of a two-dimensional square network material composed of cadmium(II) and 4,4'-bipyridine. J. Am. Chem. Soc. 116, 1151–1152. 10.1021/ja00082a055

[B37] Furukawa H. Cordova K. E. O’Keeffe M. Yaghi O. M. (2013). The chemistry and applications of metal-organic frameworks. Science 341, 1230444. 10.1126/science.1230444 23990564

[B38] Furukawa H. Gándara F. Zhang Y.-B. Jiang J. Queen W. L. Hudson M. R. (2014). Water adsorption in porous metal–organic frameworks and related materials. J. Am. Chem. Soc. 136, 4369–4381. 10.1021/ja500330a 24588307

[B39] Gao Y. Wang L. Zhou A. Li Z. Chen J. Bala H. (2015). Hydrothermal synthesis of TiO_2_/Ti_3_C_2_ nanocomposites with enhanced photocatalytic activity. Mater. Lett. 150, 62–64. 10.1016/j.matlet.2015.02.135

[B40] Gogotsi Y. Anasori B. (2019). The rise of MXenes. ACS Publications, 8491–8494. 10.1021/acsnano.9b06394 31454866

[B41] Gonuguntla S. Vennapoosa C. S. Abraham B. M. Sainath A. V. S. Pal U. (2023). Charge transfer-regulated bimetallic ZnCd-ZIF-8/Graphene oxide hybrid nanostructures for solar hydrogen generation. ACS Appl. Nano Mater. 7, 18146–18156. 10.1021/acsanm.3c02870

[B42] Habisreutinger S. N. Schmidt‐Mende L. Stolarczyk J. K. (2013). Photocatalytic reduction of CO_2_ on TiO_2_ and other semiconductors. Angew. Chem. Int. Ed. 52, 7372–7408. 10.1002/anie.201207199 23765842

[B43] Halim J. Palisaitis J. Lu J. Thörnberg J. Moon E. J. Precner M. (2018). Synthesis of two-dimensional Nb1.33C (MXene) with randomly distributed vacancies by etching of the quaternary solid solution (Nb2/3Sc1/3)2AlC MAX phase. ACS Appl. Nano Mater. 1, 2455–2460. 10.1021/acsanm.8b00332

[B44] Halim J. Persson I. Moon E. J. Kühne P. Darakchieva V. Persson P. O. Å. (2019). Electronic and optical characterization of 2D Ti_2_C and Nb_2_C (MXene) thin films. J. Phys. Condens. Matter 31, 165301. 10.1088/1361-648x/ab00a2 30669136

[B45] Han F. Luo S. Xie L. Zhu J. Wei W. Chen X. (2019). Boosting the yield of MXene 2D sheets via a facile hydrothermal-assisted intercalation. ACS Appl. Mater. and Interfaces 11, 8443–8452. 10.1021/acsami.8b22339 30697996

[B46] Han X. An L. Hu Y. Li Y. Hou C. Wang H. (2020). Ti_3_C_2_ MXene-derived carbon-doped TiO_2_ coupled with g-C_3_N_4_ as the visible-light photocatalysts for photocatalytic H_2_ generation. Appl. Catal. B 265, 118539. 10.1016/j.apcatb.2019.118539

[B47] Hong L. Klie R. F. Öğüt S. (2016). First-principles study of size- and edge-dependent properties of MXene nanoribbons. Phys. Rev. B 93, 115412. 10.1103/physrevb.93.115412

[B48] Hong L.-f. Guo R.-t. Yuan Y. Ji X.-y. Li Z.-s. Lin Z.-d. (2020b). Recent progress of two-dimensional MXenes in photocatalytic applications: a review. Mater. Today Energy 18, 100521. 10.1016/j.mtener.2020.100521

[B49] Hong W. Wyatt B. C. Nemani S. K. Anasori B. (2020a). Double transition-metal MXenes: atomistic design of two-dimensional carbides and nitrides. MRS Bull. 45, 850–861. 10.1557/mrs.2020.251

[B50] Howarth A. J. Liu Y. Li P. Li Z. Wang T. C. Hupp J. T. (2016). Chemical, thermal and mechanical stabilities of metal–organic frameworks. Nat. Rev. Mater. 1, 15018. 10.1038/natrevmats.2015.18

[B51] Huang K. Li Z. Lin J. Han G. Huang P. (2018). Two-dimensional transition metal carbides and nitrides (MXenes) for biomedical applications. Chem. Soc. Rev. 47, 5109–5124. 10.1039/c7cs00838d 29667670

[B52] Huang S. Long Y. Ruan S. Zeng Y. J. (2019). Enhanced photocatalytic CO_2_ reduction in defect-engineered Z-scheme WO_3-x_/g-C_3_N_4_ heterostructures. ACS Omega 4, 15593–15599. 10.1021/acsomega.9b01969 31572860 PMC6761746

[B53] Huang X.-C. Lin Y.-Y. Zhang J.-P. Chen X.-M. (2006). Ligand-directed strategy for zeolite-type metal–organic frameworks: zinc(II) imidazolates with unusual zeolitic topologies. Angew. Chem. Int. Ed. 45, 1557–1559. 10.1002/anie.200503778 16440383

[B54] Ibragimova R. Erhart P. Rinke P. Komsa H.-P. (2021). Surface functionalization of 2D MXenes: trends in distribution, composition, and electronic properties. J. Phys. Chem. Lett. 12, 2377–2384. 10.1021/acs.jpclett.0c03710 33657317 PMC8041312

[B55] Im J. K. Sohn E. J. Kim S. Jang M. Son A. Zoh K.-D. (2021). Review of MXene-based nanocomposites for photocatalysis. Chemosphere 270, 129478. 10.1016/j.chemosphere.2020.129478 33418219

[B56] Ivanovskii A. L. Enyashin A. N. (2013). Graphene-like transition-metal nanocarbides and nanonitrides. Russ. Chem. Rev. 82, 735–746. 10.1070/rc2013v082n08abeh004398

[B57] Jacobs C. B. Vickrey T. L. Venton B. J. (2011). Functional groups modulate the sensitivity and electron transfer kinetics of neurochemicals at carbon nanotube modified microelectrodes. Analyst 136, 3557–3565. 10.1039/c0an00854k 21373669 PMC4169050

[B58] Jalil A. Zafar Ilyas S. Agathopoulos S. Qureshi A. Ahmed I. Zhao T. (2021). 2D CoGeSe3 monolayer as a visible-light photocatalyst with high carrier mobility: theoretical prediction. Appl. Surf. Sci. 565, 150588. 10.1016/j.apsusc.2021.150588

[B59] Jeon J. Yang Y. Choi H. Park J.-H. Lee B. H. Lee S. (2020). MXenes for future nanophotonic device applications. Nanophotonics 9, 1831–1853. 10.1515/nanoph-2020-0060

[B60] Jiang X. Kuklin A. V. Baev A. Ge Y. Ågren H. Zhang H. (2020). Two-dimensional MXenes: from morphological to optical, electric, and magnetic properties and applications. Phys. Rep. 848, 1–58. 10.1016/j.physrep.2019.12.006

[B61] Jiang X. Liu S. Liang W. Luo S. He Z. Ge Y. (2018). Broadband nonlinear photonics in few-layer MXene Ti3C2Tx (T = F, O, or OH). Laser and Photonics Rev. 12, 1700229. 10.1002/lpor.201700229

[B62] Jiang X. Ren X. Chen R. Ma F. He W. Zhang T. (2023). Cobalt(II)-complex modified Ag electrode for efficient and selective electrochemical reduction of CO_2_ to CO. J. Electroanal. Chem. 949, 117860. 10.1016/j.jelechem.2023.117860

[B63] Jiang X. Sun S. Ren X. Ma F. Wen Y. He W. (2024). Au modified TiO_2_ nanowires prepared by photodeposition for selective and efficient electrochemical reduction of CO_2_ . Int. J. Hydrogen Energy 59, 168–175. 10.1016/j.ijhydene.2024.02.007

[B64] Jiao L. Seow J. Y. R. Skinner W. S. Wang Z. U. Jiang H.-L. (2019). Metal–organic frameworks: structures and functional applications. Mater. Today 27, 43–68. 10.1016/j.mattod.2018.10.038

[B65] Karthik P. Pandikumar A. Preeyanghaa M. Kowsalya M. Neppolian B. (2017). Amino-functionalized MIL-101(Fe) metal-organic framework as a viable fluorescent probe for nitroaromatic compounds. Microchim. Acta 184, 2265–2273. 10.1007/s00604-017-2215-2

[B66] Karthik P. Shaheer A. R. M. Vinu A. Neppolian B. (2020). Amine functionalized metal-organic framework coordinated with transition metal ions: d-d transition enhanced optical absorption and role of transition metal sites on solar light driven H(2) production. Small 16, e1902990. 10.1002/smll.201902990 31724829

[B67] Karthik P. Vinoth R. Zhang P. Choi W. Balaraman E. Neppolian B. (2018). π–π interaction between metal–organic framework and reduced graphene oxide for visible-light photocatalytic H2 production. ACS Appl. Energy Mater. 1, 1913–1923. 10.1021/acsaem.7b00245

[B68] Kayali E. VahidMohammadi A. Orangi J. Beidaghi M. (2018). Controlling the dimensions of 2D MXenes for ultrahigh-rate pseudocapacitive energy storage. ACS Appl. Mater. and Interfaces 10, 25949–25954. 10.1021/acsami.8b07397 30044609

[B69] Khan A. A. Tahir M. (2019). Recent advancements in engineering approach towards design of photo-reactors for selective photocatalytic CO_2_ reduction to renewable fuels. J. CO_2_ Util. 29, 205–239. 10.1016/j.jcou.2018.12.008

[B70] Khan A. A. Tahir M. (2021). Well-designed 2D/2D Ti_3_C_2_T_A/R_ MXene coupled g-C_3_N_4_ heterojunction with *in-situ* growth of anatase/rutile TiO_2_ nucleates to boost photocatalytic dry-reforming of methane (DRM) for syngas production under visible light. Appl. Catal. B 285, 119777. 10.1016/j.apcatb.2020.119777

[B71] Khazaei M. Arai M. Sasaki T. Chung C.-Y. Venkataramanan N. S. Estili M. (2012). Novel electronic and magnetic properties of two-dimensional transition metal carbides and nitrides. Adv. Funct. Mater. 23, 2185–2192. 10.1002/adfm.201202502

[B72] Khazaei M. Ranjbar A. Arai M. Sasaki T. Yunoki S. (2017). Electronic properties and applications of MXenes: a theoretical review. J. Mater. Chem. C 5, 2488–2503. 10.1039/c7tc00140a

[B73] Kong F. He X. Liu Q. Qi X. Zheng Y. Wang R. (2018). Improving the electrochemical properties of MXene Ti_3_C_2_ multilayer for Li-ion batteries by vacuum calcination. Electrochimica Acta 265, 140–150. 10.1016/j.electacta.2018.01.196

[B74] Lee Y. Cho S. B. Chung Y.-C. (2014). Tunable indirect to direct band gap transition of monolayer Sc_2_CO_2_ by the strain effect. ACS Appl. Mater. and Interfaces 6, 14724–14728. 10.1021/am504233d 25105743

[B75] Li D. Xu H.-Q. Jiao L. Jiang H.-L. (2019a). Metal-organic frameworks for catalysis: state of the art, challenges, and opportunities. EnergyChem 1, 100005. 10.1016/j.enchem.2019.100005

[B76] Li H. Eddaoudi M. O'Keeffe M. Yaghi O. M. (1999). Design and synthesis of an exceptionally stable and highly porous metal-organic framework. Nature 402, 276–279. 10.1038/46248

[B77] Li J.-R. Kuppler R. J. Zhou H.-C. (2009). Selective gas adsorption and separation in metal–organic frameworks. Chem. Soc. Rev. 38, 1477–1504. 10.1039/b802426j 19384449

[B78] Li J.-R. Ma Y. McCarthy M. C. Sculley J. Yu J. Jeong H.-K. (2011). Carbon dioxide capture-related gas adsorption and separation in metal-organic frameworks. Coord. Chem. Rev. 255, 1791–1823. 10.1016/j.ccr.2011.02.012

[B79] Li N. Liu J. Liu J. J. Dong L. Z. Xin Z. F. Teng Y. L. (2019b). Adenine components in biomimetic metal-organic frameworks for efficient CO_2_ photoconversion. Angew. Chem. Int. Ed. Engl. 58, 5226–5231. 10.1002/anie.201814729 30656814

[B80] Li S. Wu F. Lin R. Wang J. Li C. Li Z. (2022). Enabling photocatalytic hydrogen production over Fe-based MOFs by refining band structure with dye sensitization. Chem. Eng. J. 429, 132217. 10.1016/j.cej.2021.132217

[B81] Li Y. Liu Y. Wang Z. Wang P. Zheng Z. Cheng H. (2021). *In-situ* growth of Ti_3_C_2_@MIL-NH_2_ composite for highly enhanced photocatalytic H_2_ evolution. Chem. Eng. J. 411, 128446. 10.1016/j.cej.2021.128446

[B82] Li Z. Wu Y. (2019). 2D early transition metal carbides (MXenes) for catalysis. Small 15, 1804736. 10.1002/smll.201804736 30672102

[B83] Liao W. M. Zhang J. H. Wang Z. Lu Y. L. Yin S. Y. Wang H. P. (2018). Semiconductive amine-functionalized Co(II)-MOF for visible-light-driven hydrogen evolution and CO_2_ reduction. Inorg. Chem. 57, 11436–11442. 10.1021/acs.inorgchem.8b01265 30152695

[B84] Lim K. R. G. Handoko A. D. Nemani S. K. Wyatt B. Jiang H.-Y. Tang J. (2020). Rational design of two-dimensional transition metal carbide/nitride (MXene) hybrids and nanocomposites for catalytic energy storage and conversion. ACS Nano 14, 10834–10864. 10.1021/acsnano.0c05482 32790329

[B85] Lin S. Song Z. Che G. Ren A. Li P. Liu C. (2014). Adsorption behavior of metal–organic frameworks for methylene blue from aqueous solution. Microporous mesoporous Mater. 193, 27–34. 10.1016/j.micromeso.2014.03.004

[B86] Linsebigler A. L. Lu G. Yates J. T. (1995). Photocatalysis on TiO_2_ surfaces: principles, mechanisms, and selected results. Chem. Rev. 95, 735–758. 10.1021/cr00035a013

[B87] Liu H.-J. Dong B. (2021). Recent advances and prospects of MXene-based materials for electrocatalysis and energy storage. Mater. Today Phys. 20, 100469. 10.1016/j.mtphys.2021.100469

[B88] Liu M. Zhou L. Luo X. Wan C. Xu L. (2020a). Recent advances in noble metal catalysts for hydrogen production from ammonia borane. Catalysts 10, 788. 10.3390/catal10070788

[B89] Liu Q. Tan X. Wang S. Ma F. Znad H. Shen Z. (2019). MXene as a non-metal charge mediator in 2D layered CdS@ Ti_3_C_2_@TiO_2_ composites with superior Z-scheme visible light-driven photocatalytic activity. Environ. Sci. Nano 6, 3158–3169. 10.1039/c9en00567f

[B90] Liu S. Zhang C. Sun Y. Chen Q. He L. Zhang K. (2020b). Design of metal-organic framework-based photocatalysts for hydrogen generation. Coord. Chem. Rev. 413, 213266. 10.1016/j.ccr.2020.213266

[B91] Liu X. Shan Y. Zhang S. Kong Q. Pang H. (2022). Application of metal organic framework in wastewater treatment. Green Energy and Environ. 8, 698–721. 10.1016/j.gee.2022.03.005

[B92] Loiseau T. Serre C. Huguenard C. Fink G. Taulelle F. Henry M. (2004). A rationale for the large breathing of the porous aluminum terephthalate (MIL-53) upon hydration. Chem. - A Eur. J. 10, 1373–1382. 10.1002/chem.200305413 15034882

[B93] Long R. Yu Z. Tan Q. Feng X. Zhu X. Li X. (2021). Ti_3_C_2_ MXene/NH_2_-MIL-88B(Fe): research on the adsorption kinetics and photocatalytic performance of an efficient integrated photocatalytic adsorbent. Appl. Surf. Sci. 570, 151244. 10.1016/j.apsusc.2021.151244

[B94] Lu W. Wei Z. Gu Z.-Y. Liu T.-F. Park J. Park J. (2014). Tuning the structure and function of metal–organic frameworks via linker design. Chem. Soc. Rev. 43, 5561–5593. 10.1039/c4cs00003j 24604071

[B95] Maeda K. (2011). Photocatalytic water splitting using semiconductor particles: history and recent developments. J. Photochem. Photobiol. C Photochem. Rev. 12, 237–268. 10.1016/j.jphotochemrev.2011.07.001

[B96] Naguib M. Kurtoglu M. Presser V. Lu J. Niu J. Heon M. (2011). Two-dimensional nanocrystals produced by exfoliation of Ti_3_AlC_2_ . Adv. Mater. 23, 4248–4253. 10.1002/adma.201102306 21861270

[B97] Najam T. Shah S. S. A. Peng L. Javed M. S. Imran M. Zhao M.-Q. (2022). Synthesis and nano-engineering of MXenes for energy conversion and storage applications: recent advances and perspectives. Coord. Chem. Rev. 454, 214339. 10.1016/j.ccr.2021.214339

[B98] Nguyen J. G. Cohen S. M. (2010). Moisture-resistant and superhydrophobic Metal−Organic frameworks obtained via postsynthetic modification. J. Am. Chem. Soc. 132, 4560–4561. 10.1021/ja100900c 20232871 PMC2860283

[B99] Papadopoulou K. Chroneos A. Parfitt D. Christopoulos S.-R. G. (2020). A perspective on MXenes: their synthesis, properties, and recent applications. J. Appl. Phys. 128, 170902. 10.1063/5.0021485

[B100] Park K. S. Ni Z. Côté A. P. Choi J. Y. Huang R. Uribe-Romo F. J. (2006). Exceptional chemical and thermal stability of zeolitic imidazolate frameworks. Proc. Natl. Acad. Sci. 103, 10186–10191. 10.1073/pnas.0602439103 16798880 PMC1502432

[B101] Perry Iv J. J. Perman J. A. Zaworotko M. J. (2009). Design and synthesis of metal–organic frameworks using metal–organic polyhedra as supermolecular building blocks. Chem. Soc. Rev. 38, 1400. 10.1039/b807086p 19384444

[B102] Persson I. el Ghazaly A. Tao Q. Halim J. Kota S. Darakchieva V. (2018). Tailoring structure, composition, and energy storage properties of MXenes from selective etching of in-plane, chemically ordered MAX phases. Small 14, 1703676. 10.1002/smll.201703676 29611285

[B103] Qin T. Wang Z. Wang Y. Besenbacher F. Otyepka M. Dong M. (2021). Recent progress in emerging two-dimensional transition metal carbides. Nano-Micro Lett. 13, 183. 10.1007/s40820-021-00710-7 PMC8379312 34417663

[B104] Reddy C. V. Reddy K. R. Harish V. V. N. Shim J. Shankar M. V. Shetti N. P. (2020). Metal-organic frameworks (MOFs)-based efficient heterogeneous photocatalysts: synthesis, properties and its applications in photocatalytic hydrogen generation, CO_2_ reduction and photodegradation of organic dyes. Int. J. Hydrogen Energy 45, 7656–7679. 10.1016/j.ijhydene.2019.02.144

[B105] Reddy D. A. Kim Y. Gopannagari M. Kumar D. P. Kim T. K. (2021). Recent advances in metal–organic framework-based photocatalysts for hydrogen production. Sustain. Energy and Fuels 5, 1597–1618. 10.1039/c9se00749k

[B106] Ren X. Liao G. Li Z. Qiao H. Zhang Y. Yu X. (2021). Two-dimensional MOF and COF nanosheets for next-generation optoelectronic applications. Coord. Chem. Rev. 435, 213781. 10.1016/j.ccr.2021.213781

[B107] Ren X. Liu Z. Zhang T. Jiang X. Fang Q. Li Y. (2024). Review on two-dimensional metal–organic frameworks for biological sensing: current challenges and new frontiers. J. Mater. Sci. 59, 10724–10743. 10.1007/s10853-024-09818-8

[B108] Ronchi R. M. Arantes J. T. Santos S. F. (2019). Synthesis, structure, properties and applications of MXenes: current status and perspectives. Ceram. Int. 45, 18167–18188. 10.1016/j.ceramint.2019.06.114

[B109] Senkovska I. Kaskel S. (2014). Ultrahigh porosity in mesoporous MOFs: promises and limitations. Chem. Commun. 50, 7089–7098. 10.1039/c4cc00524d 24722662

[B110] Sharma K. Hasija V. Patial S. Singh P. Nadda A. Thakur S. (2022b). Recent progress on MXenes and MOFs hybrids: structure, synthetic strategies and catalytic water splitting. Int. J. Hydrogen Energy 48, 6560–6574. 10.1016/j.ijhydene.2022.01.004

[B111] Sharma K. Hasija V. Patial S. Singh P. Nguyen V.-H. Nadda A. K. (2022c). Recent progress on MXenes and MOFs hybrids: structure, synthetic strategies and catalytic water splitting. Int. J. Hydrogen Energy 48, 6560–6574. 10.1016/j.ijhydene.2022.01.004

[B112] Sharma S. K. Kumar A. Sharma G. Vo D.-V. N. García-Peñas A. Moradi O. (2022a). MXenes based nano-heterojunctions and composites for advanced photocatalytic environmental detoxification and energy conversion: a review. Chemosphere 291, 132923. 10.1016/j.chemosphere.2021.132923 34813851

[B113] Sherryna A. Tahir M. (2021). Role of Ti_3_C_2_ MXene as prominent Schottky barriers in driving hydrogen production through photoinduced water splitting: a comprehensive review. ACS Appl. Energy Mater. 4, 11982–12006. 10.1021/acsaem.1c02241

[B114] Sherryna A. Tahir M. (2022a). Role of surface morphology and terminating groups in titanium carbide MXenes (Ti_3_C_2_T_x_) cocatalysts with engineering aspects for modulating solar hydrogen production: a critical review. Chem. Eng. J. 433, 134573. 10.1016/j.cej.2022.134573

[B115] Sherryna A. Tahir M. (2022b). Role of surface morphology and terminating groups in titanium carbide MXenes (Ti_3_C_2_T_x_) cocatalysts with engineering aspects for modulating solar hydrogen production: a critical review. Chem. Eng. J. 433, 134573. 10.1016/j.cej.2022.134573

[B116] Sherryna A. Tahir M. Nabgan W. (2021). Recent advancements of layered double hydroxide heterojunction composites with engineering approach towards photocatalytic hydrogen production: a review. Int. J. Hydrogen Energy 47, 862–901. 10.1016/j.ijhydene.2021.10.099

[B117] Shi Z. Khaledialidusti R. Malaki M. Zhang H. (2021). MXene-based materials for solar cell applications. Nanomaterials 11, 3170. 10.3390/nano11123170 34947518 PMC8707056

[B118] Shkrob I. A. Sauer M. C. (2004). Hole scavenging and photo-stimulated recombination of electron− hole pairs in aqueous TiO_2_ nanoparticles. J. Phys. Chem. B 108, 12497–12511. 10.1021/jp047736t

[B119] Si C. Jin K.-H. Zhou J. Sun Z. Liu F. (2016). Large-gap quantum spin Hall state in MXenes: d-band topological order in a triangular lattice. Nano Lett. 16, 6584–6591. 10.1021/acs.nanolett.6b03118 27622311

[B120] Song Z. Song S. Zhang W. Han H. Wei K. Liu D. (2024). The unique Z-scheme g-C_3_N_4_/Ti_3_C_2_/Sn-Bi-MOF photocatalyst for enhancing CO_2_ reduction activity. Fuel 366, 131154. 10.1016/j.fuel.2024.131154

[B121] Suman S. (2018). Hybrid nuclear-renewable energy systems: a review. J. Clean. Prod. 181, 166–177. 10.1016/j.jclepro.2018.01.262

[B122] Tahir M. (2021a). Binary Ni_2_P/Ti_3_C_2_ multilayer cocatalyst anchored TiO_2_ nanocomposite with etchant/oxidation grown TiO_2_ NPs for enhancing photocatalytic H_2_ production. Energy and Fuels 35, 14197–14211. 10.1021/acs.energyfuels.1c01340

[B123] Tahir M. (2021b). Investigating the influential effect of etchant time in constructing 2D/2D HCN/MXene heterojunction with controlled growth of TiO_2_ NPs for stimulating photocatalytic H_2_ production. Energy and Fuels 35, 6807–6822. 10.1021/acs.energyfuels.1c00204

[B124] Tahir M. (2024). Synergistic effect of the V_2_CTx MXene@V_2_O_5_/TiO_2_ NP composite for stimulating photocatalytic CO_2_ reduction through bireforming of methanol to produce CO and CH_4_ . Energy and Fuels 38, 10183–10202. 10.1021/acs.energyfuels.3c05215

[B125] Tahir M. Ajiwokewu B. Bankole A. A. Ismail O. Al-Amodi H. Kumar N. (2023a). MOF based composites with engineering aspects and morphological developments for photocatalytic CO_2_ reduction and hydrogen production: a comprehensive review. J. Environ. Chem. Eng. 11, 109408. 10.1016/j.jece.2023.109408

[B126] Tahir M. Khan A. A. Tasleem S. Mansoor R. Sherryna A. Tahir B. (2023b). Recent advances in titanium carbide MXene-based nanotextures with influential effect of synthesis parameters for solar CO_2_ reduction and H_2_ production: a critical review. J. Energy Chem. 76, 295–331. 10.1016/j.jechem.2022.09.046

[B127] Tahir M. Fan W. K. Tahir B. (2021a). MOF-based catalysts for production of value-added fine chemicals from carbon dioxide, Metal−Organic frameworks for carbon capture and energy. ACS Publications, 155–171.

[B128] Tahir M. Sherryna A. Zakaria Z. Y. (2021b). Facile synthesis of MAX modified graphitic carbon nitride nanocomposite for stimulating hydrogen production through photocatalytic water splitting. Chem. Eng. Trans. 89, 571–576.

[B129] Tahir M. Tahir B. (2020). 2D/2D/2D O-C_3_N_4_/Bt/Ti_3_C_2_Tx heterojunction with novel MXene/clay multi-electron mediator for stimulating photo-induced CO_2_ reforming to CO and CH_4_ . Chem. Eng. J. 400, 125868. 10.1016/j.cej.2020.125868

[B130] Tasleem S. Tahir M. Zakaria Z. Y. (2020). Fabricating structured 2D Ti_3_AlC_2_ MAX dispersed TiO_2_ heterostructure with Ni_2_P as a cocatalyst for efficient photocatalytic H_2_ production. J. Alloys Compd. 842, 155752. 10.1016/j.jallcom.2020.155752

[B131] Tian P. He X. Zhao L. Li W. Fang W. Chen H. (2018). Enhanced charge transfer for efficient photocatalytic H2 evolution over UiO-66-NH_2_ with annealed Ti_3_C_2_T_x_ MXenes. Int. J. Hydrogen Energy 44, 788–800. 10.1016/j.ijhydene.2018.11.016

[B132] Tian P. He X. Zhao L. Li W. Fang W. Chen H. (2019a). Enhanced charge transfer for efficient photocatalytic H_2_ evolution over UiO-66-NH_2_ with annealed Ti_3_C_2_T_x_ MXenes. Int. J. Hydrogen Energy 44, 788–800. 10.1016/j.ijhydene.2018.11.016

[B133] Tian P. He X. Zhao L. Li W. Fang W. Chen H. (2019b). Ti3C2 nanosheets modified Zr-MOFs with Schottky junction for boosting photocatalytic HER performance. Sol. Energy 188, 750–759. 10.1016/j.solener.2019.06.060

[B134] Tranchemontagne D. J. Mendoza-Cortés J. L. O’Keeffe M. Yaghi O. M. (2009). Secondary building units, nets and bonding in the chemistry of metal–organic frameworks. Chem. Soc. Rev. 38, 1257–1283. 10.1039/b817735j 19384437

[B135] Urbankowski P. Anasori B. Hantanasirisakul K. Yang L. Zhang L. Haines B. (2017). 2D molybdenum and vanadium nitrides synthesized by ammoniation of 2D transition metal carbides (MXenes). Nanoscale 9, 17722–17730. 10.1039/c7nr06721f 29134998

[B136] Wang Q. Gao Q. Al-Enizi A. M. Nafady A. Ma S. (2020). Recent advances in MOF-based photocatalysis: environmental remediation under visible light. Inorg. Chem. Front. 7, 300–339. 10.1039/c9qi01120j

[B137] Wong Z. M. Tan T. L. Yang S.-W. Xu G. Q. (2018). Enhancing the photocatalytic performance of MXenes via stoichiometry engineering of their electronic and optical properties. ACS Appl. Mater. and Interfaces 10, 39879–39889. 10.1021/acsami.8b14325 30353717

[B138] Wu H. Almalki M. Xu X. Lei Y. Ming F. Mallick A. (2019). MXene derived metal–organic frameworks. J. Am. Chem. Soc. 141, 20037–20042. 10.1021/jacs.9b11446 31825615

[B139] Xie J. Yang C. Duan M. Tang J. Wang Y. Wang H. (2018). Amorphous NiP as cocatalyst for photocatalytic water splitting. Ceram. Int. 44, 5459–5465. 10.1016/j.ceramint.2017.12.179

[B140] Xu Q. Sun Y. Lv T. Liu H. (2023). Selective CO_2_ photoreduction into CO over Ti_3_C_2_ quantum dots decorated NH_2_-MIL-101(Fe) heterostructures. J. Alloys Compd. 954, 170088. 10.1016/j.jallcom.2023.170088

[B141] Xuan W. Zhu C. Liu Y. Cui Y. (2012). Mesoporous metal–organic framework materials. Chem. Soc. Rev. 41, 1677–1695. 10.1039/c1cs15196g 22008884

[B142] Yang C. Kaipa U. Mather Q. Z. Wang X. Nesterov V. Venero A. F. (2011). Fluorous metal–organic frameworks with superior adsorption and hydrophobic properties toward oil spill cleanup and hydrocarbon storage. J. Am. Chem. Soc. 133, 18094–18097. 10.1021/ja208408n 21981413

[B143] Ying G. Dillon A. D. Fafarman A. T. Barsoum M. W. (2017). Transparent, conductive solution processed spincast 2D Ti_2_CT_x_ (MXene) films. Mater. Res. Lett. 5, 391–398. 10.1080/21663831.2017.1296043

[B144] You Z. Liao Y. Li X. Fan J. Xiang Q. (2021). State-of-the-art recent progress in MXene-based photocatalysts: a comprehensive review. Nanoscale 13, 9463–9504. 10.1039/d1nr02224e 34028480

[B145] Yu L. Liu B. Wang Y. Yu F. Ma J. (2021). Recent progress on MXene-Derived material and its’ application in energy and environment. J. Power Sources 490, 229250. 10.1016/j.jpowsour.2020.229250

[B146] Zhang B. Zhang S.-X. Yao R. Wu Y.-H. Qiu J.-S. (2021). Progress and prospects of hydrogen production: opportunities and challenges. J. Electron. Sci. Technol. 19, 100080. 10.1016/j.jnlest.2021.100080

[B147] Zhang T. Ren X. Mo S. Cao W. Zhou C. Ma F. (2024). Modulating Fe/P ratios in Fe-P alloy through smelting reduction for long-term electrocatalytic overall water splitting. J. Mater. Sci. and Technol. 199, 66–74. 10.1016/j.jmst.2024.02.037

[B148] Zhao J.-H. Liu L.-W. Li K. Li T. Liu F.-T. (2019). Conductive Ti_3_C_2_ and MOF-derived CoS_x_ boosting the photocatalytic hydrogen production activity of TiO_2_ . CrystEngComm 21, 2416–2421. 10.1039/c8ce02050g

[B149] Zhao X. Lu Z. Zhang Y. Zhou M. Xu S. Li Z. (2022). A review of recent progress in modified metal–organic frameworks as photocatalysts. J. Mater. Sci. Mater. Electron. 33, 4737–4754. 10.1007/s10854-022-07717-9

[B150] Zhou H.-C. J. Kitagawa S. (2014). Metal–organic frameworks (MOFs). Chem. Soc. Rev. 43, 5415–5418. 10.1039/c4cs90059f 25011480

[B151] Zhou Y. Ren X. Wang X. Mao J. Zhang H. Wang J. (2024). Promoting CuO/Cu(OH)_2_ for electrocatalytic reduction of CO_2_ to HCOOH: the study on pyridine-modified surface active sites. Mol. Catal. 556, 113929. 10.1016/j.mcat.2024.113929

[B152] Zong H. Qi R. Yu K. Zhu Z. (2021). Ultrathin Ti2NTx MXene-wrapped MOF-derived CoP frameworks towards hydrogen evolution and water oxidation. Electrochimica Acta 393, 139068. 10.1016/j.electacta.2021.139068

